# IL33-induced lipid droplet formation in mature low-density neutrophils drives colorectal cancer liver metastasis

**DOI:** 10.1038/s41423-025-01365-9

**Published:** 2025-11-10

**Authors:** Yuchen Zhang, Suyue Yu, Dina Yeernuer, Wangyi Liu, Zhuoqing Xu, Wenqing Feng, Zeping Lv, Xuanhao Liu, Peiqi Tan, Minhua Zheng, Yaping Zong, Aiguo Lu, Jingkun Zhao

**Affiliations:** 1 Department of General Surgery, Ruijin Hospital, Shanghai Jiaotong University School of Medicine, Shanghai, China; 2 Shanghai Minimally Invasive Surgery Center, Ruijin Hospital, Shanghai Jiaotong University School of Medicine, Shanghai, China

**Keywords:** metabolic reprogramming, mature low-density neutrophils, lipid droplets, liver metastasis, colorectal cancer, Colorectal cancer, Cancer microenvironment, Interleukins, Cancer metabolism, Cancer immunotherapy

## Abstract

The microenvironment of distant organs affects the colonization and growth of disseminated tumor cells. It remains unclear how tumor-associated neutrophils are influenced by the microenvironment of distant organs. Here, we demonstrate that mature low-density neutrophils in colorectal cancer patients abnormally accumulate neutral lipids and induce the reactivation of dormant tumor cells, a process regulated by hepatic stellate cells. Mechanistically, activated hepatic stellate cells increased DGAT1/2-dependent lipid droplet synthesis in low-density neutrophils through the secretion of IL33, thereby maintaining the survival and immunosuppressive function of these neutrophils. The uptake of lipids from lipid-laden low-density neutrophils drives dormant tumor cell reactivation through the potentiation of β-oxidation and the stimulation of protumorigenic eicosanoid synthesis. In mouse models, targeting IL33 blocked neutrophil lipid synthesis, decreased the colonization of colorectal cancer cells in the liver, and enhanced the efficacy of immunotherapy. Overall, our study revealed that lipid accumulation in mature low-density neutrophils regulates the growth of dormant tumor cells and antitumor immunity to facilitate colorectal cancer liver metastasis. Targeting IL33 could be a promising therapeutic approach for colorectal cancer liver metastases.

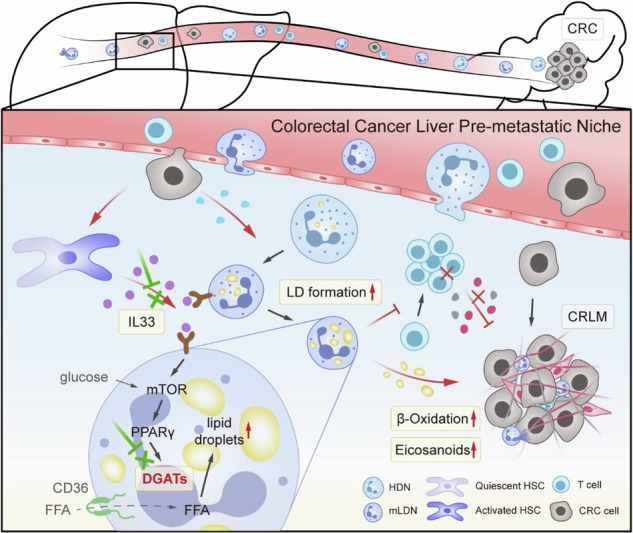

## Introduction

Metastasis is the main factor leading to death in people with cancer [1]. During the process of metastasis [2–5], the colonization of distant organs with disseminated tumor cells (DTCs), which requires the right population of tumor cells and a suitable microenvironment, is an “inefficient” process [6]. In the bloodstream, DTCs enter a state of cell cycle arrest, also referred to as a dormant state, to sustain their survival and evade immune surveillance [7]. To regrow and proliferate in distant organs as visible lesions on imaging, DTCs must exit this dormant state. The colonization of tumor cells is related to interactions among DTCs, immune cells and stromal cells in the premetastatic niche (PMN) [8].

Prior to tumor metastasis, infiltrating protumor neutrophils appear in distant organs in response to the tumor and its derived cytokines [9]. In the premetastatic lung microenvironment, neutrophils are recruited by HSP70, a soluble mediator secreted by tumor cells that induces CXCR2-dependent chemokines and promotes the progression of lung metastasis [10]. Low-density neutrophils (LDNs) are a special subpopulation of neutrophils with a density similar to that of monocytes. Individuals with alcoholic hepatitis have a unique LDN population, which is generated from high-density neutrophils and has a reduced phagocytic capacity, homing capacity and clearance by macrophage efferocytosis, resulting in a functionally exhausted phenotype [11]. Research has suggested that low-density neutrophils exist in the blood circulation of colorectal cancer patients. The role of LDNs in regulating metastasis has gradually emerged. However, neutrophils in the blood have a short survival time. Although exposure to the tumor environment is crucial for extending the lifespan of neutrophils [12], the sustained role of infiltrating neutrophils in the PMN is still unknown.

Neutrophils are powered mainly by glycolysis, which makes the independence of a high sugar supply even more important [13]. However, compared with the glucose content in the blood, glucose availability in tissues may be lower. The upregulation of GLUT1 expression by tumor-infiltrating neutrophils in lung adenocarcinoma promotes tumor growth and radiation resistance [14]. In fact, our recent data indicate that LDNs induced by colorectal cancer cells accumulate large amounts of neutral lipids. Neutrophils infiltrating lung cancer cells are reportedly laden with lipids and can transfer lipids to tumor cells to promote cancer cell proliferation and the squamous transition [15]. These data emphasize the importance of maintaining LDN protumor function through lipid metabolism pathways when glucose availability is limited. However, how LDN lipid metabolism is regulated by the PMN and affects liver metastasis in colorectal cancer patients is currently unclear.

Here, we found that LDNs in the liver metastasis microenvironment had higher levels of neutral lipids than those in the primary focus. Lipid metabolism in LDNs induced by activated hepatic stellate cells (HSCs) was found to be regulated by IL33/PPARγ/DGATs. Lipid droplets prevent the lipotoxicity of externally obtained fatty acids and maintain the ability of neutrophils to inhibit T-cell activation and induce the reactivation of dormant tumor cells. The reactivation of dormant tumor cells is mediated through the uptake of neutrophil-derived lipids coupled with increased fatty acid oxidation and eicosanoid production. The combination of anti-IL33 therapy and immunotherapy inhibited the occurrence of colorectal cancer liver metastasis. Therefore, our study elucidated the specific mechanism by which the microenvironment regulates LDN lipid metabolism to maintain its prometastatic and immunosuppressive functions and revealed the therapeutic potential of targeting neutrophil lipid accumulation combined with immunotherapy.

## Results

### Lipid storage by M-LDNs in the blood increases as the tumor stage progresses

**Fig. 1:**
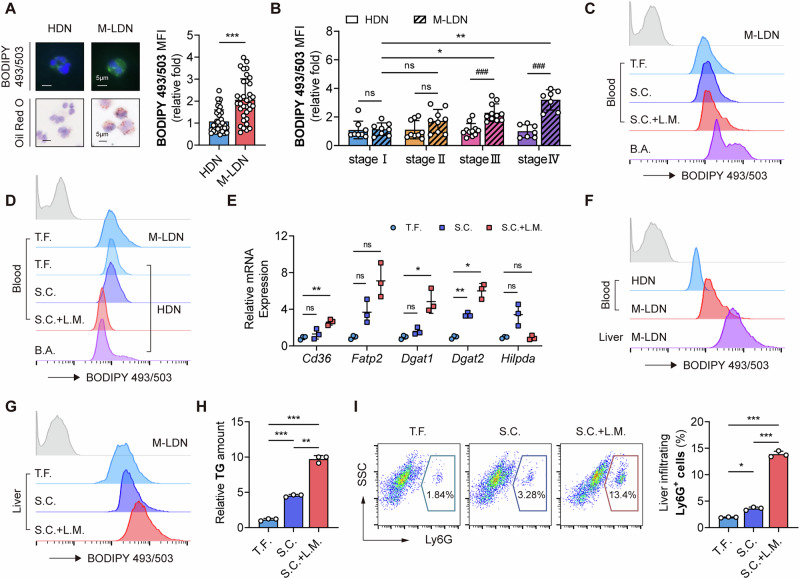
Lipid accumulation in mature low-density neutrophils in the blood increases in advanced colorectal cancer. **A** Lipid staining of neutrophils from colorectal cancer patients detected by BODIPY 493/503 and Oil Red O. Lipid quantification by flow cytometry (*n* = 35). Scale bar, 5 μm. **B** Intracellular lipids of patient neutrophils in different tumor stages quantified by flow cytometry (*n* = 8, 9, 11 and 7 for stages I to IV, respectively). MFI, mean fluorescence intensity. **C**, **D** Lipid levels in neutrophils from the peripheral blood and ascites of different tumor-bearing BALB/c mice (*n* = 3). T.F., tumor-free. S.C., subcutaneous. L.M., liver metastasis. B.A., bloody ascites. **E** Relative mRNA expression of genes related to lipid uptake and synthesis in M-LDNs (*n* = 3). **F** Lipid content quantification of neutrophils in the peripheral blood and liver of tumor-bearing mice (*n* = 3). **G**, **H** Lipid levels in liver-infiltrating neutrophils from different tumor-bearing BALB/c mice quantified by BODIPY 493/503 and TG levels (*n* = 3). **I** Proportion of Ly6G^+^ liver-infiltrating neutrophils in BALB/c mice at different stages (*n* = 3)

Our previous work revealed that colorectal cancer cells can promote the transformation of high-density neutrophils (HDNs) to mature low-density neutrophils (M-LDNs). M-LDNs acquire tumor-promoting and immunosuppressive functions and increase liver metastasis in patients with colorectal cancer. Tumor cells modify their energy metabolism patterns to fulfill their biological energy and synthesis requirements to sustain their survival [16]. Among these modifications, lipid metabolism reprogramming is a significant factor influencing cellular function [17]. To detect changes in the lipid metabolism of M-LDNs during the progression of CRC, we compared the lipid content of neutrophils in the peripheral blood of patients at different stages of CRC. The lipid levels in M-LDNs in peripheral blood were significantly greater than those in HDNs (Fig. 1A). Moreover, the M-LDNs presented high transcription levels of genes related to lipid uptake and synthesis (Fig. S1A). Moreover, compared with that in HDNs, the lipid content in M-LDNs in the peripheral blood of stage III and IV patients increased more significantly (Fig. 1B). As the tumors progressed, the lipids in the M-LDNs but not those in the HDNs increased (Fig. 1B). To verify the accumulation of lipids in M-LDNs during cancer occurrence and progression, we established mouse models of colorectal cancer at different stages [18]. Similarly, we found that the neutral lipid content in M-LDNs in the blood of nonmetastatic tumor-bearing mice was greater than that in M-LDNs in the blood of tumor-free mice. In addition, the neutral lipid content continued to increase during the liver metastasis stage (Fig. 1C). However, among the different models, the lipid content in HDNs did not significantly differ (Fig. 1D). We also noticed that in mice with bloody ascites and late-stage tumors, the lipid content in M-LDNs in ascites was even greater (Fig. 1C), indicating that the lipid levels in M-LDNs were correlated with tumor progression. The expression of genes related to lipid uptake and synthesis by M-LDNs in mice with liver metastasis tended to increase (Fig. 1E). Therefore, during colorectal cancer progression, M-LDNs in peripheral blood continuously accumulate neutral lipids.

### Colorectal cancer cells induce lipid accumulation in neutrophils and transition to M-LDNs

Our previous studies indicated that HDN degranulation to form M-LDNs is induced by colorectal cancer cells. We speculate that tumor cells promote neutrophil lipid enrichment while inducing a decrease in neutrophil density. The lipid content in M-LDNs that infiltrated into colorectal cancer tissue was assessed. Compared with those in the blood, tumor-infiltrating M-LDNs accumulated more neutral lipids (Fig. S1B). After HDNs were treated with CM from tumor cells (i.e., tumor-educated HDNs, teHDNs), the neutral lipid content significantly increased and reached the level observed in M-LDNs (Fig. S1C). To confirm that HDNs acquire increased lipid content through continuous oncogenic stimulation, we compared neutral lipid levels in HDNs subjected to either sustained tumor CM stimulation or withdrawal after stimulation. The results demonstrated that teHDNs maintained elevated neutral lipids under persistent tumor CM exposure, whereas lipid levels decreased upon tumor CM withdrawal (Fig. S1D-E). Oxidized lipids were present only in small amounts in tumor-infiltrating M-LDNs, and tumor CM did not increase the amount of oxidized lipids in HDNs (Fig. S1F). We then compared the oxidative lipid uptake capacity of various neutrophils. M-LDNs had the strongest ability to take up oxidative lipids. Tumor CM partially induced the uptake of oxidative lipids by HDNs. However, similar to that of tumor-infiltrating M-LDNs, the ability of HDNs to take up lipids was weaker (Fig. S1G). These findings indicate that tumor cells mainly promote the accumulation of neutral lipids rather than oxidative lipids (which may be caused by other mechanisms) in neutrophils. In addition, we compared the changes in glucose and cholesterol metabolism between teHDNs and M-LDNs. The metabolic patterns of teHDNs and M-LDNs were similar, with the expression of most glycolytic genes upregulated except for GLUT3 and most cholesterol metabolism genes downregulated (Fig. S1H). Therefore, colorectal cancer cells can induce metabolic changes in HDNs in an M-LDN pattern, and the accumulation of neutral lipids in M-LDNs can be induced by colorectal cancer cells.

### M-LDNs that infiltrate metastatic liver tumors contain more lipid droplets, a process induced by hepatic stellate cells

**Fig. 2:**
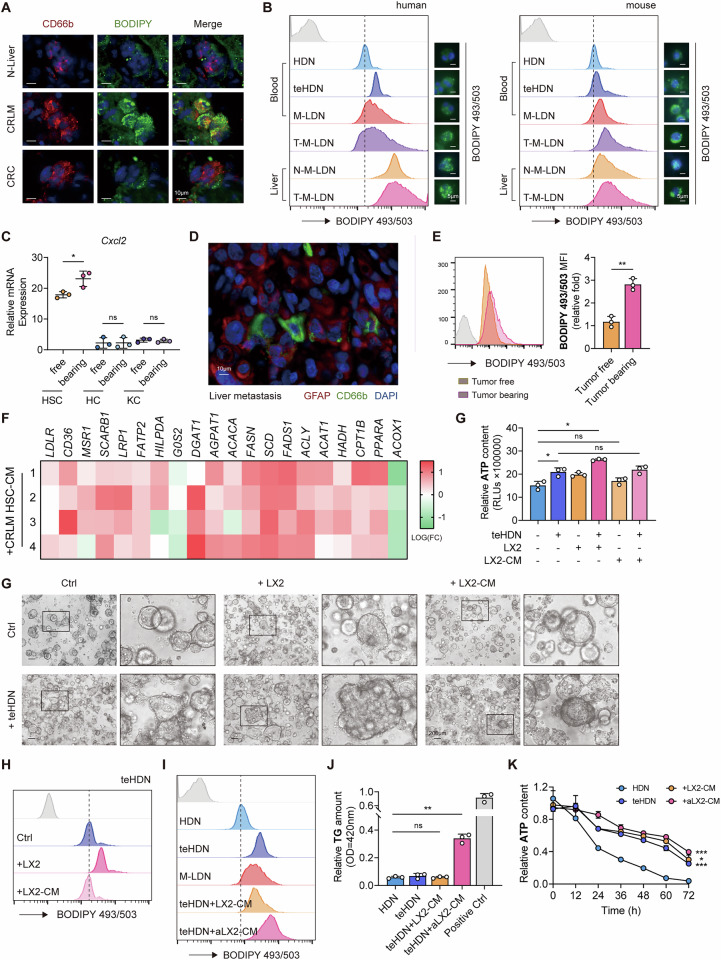
Low-density neutrophils infiltrating the metastatic liver have more lipid droplets further induced by hepatic stellate cells. **A** Immunostaining of normal liver, liver metastasis and colorectal cancer tissues showing the relative lipid content of infiltrating neutrophils. Scale bar, 10 μm. **B** Lipid levels and representative images of blood, tumor-infiltrating and liver-infiltrating neutrophils (*n* = 3). Scale bar, 5 μm. **C** Relative mRNA expression of CXCL2 in HSCs, hepatocytes (HCs) and Kupffer cells (KCs) from tumor-free and tumor-bearing BALB/c mice (*n* = 3). **D** Immunostaining of patient liver metastases showing the localization of neutrophils and HSCs. Scale bar, 10 μm. **E** Lipid levels in neutrophils cocultured with HSCs from tumor-free and tumor-bearing mice (*n* = 3). **F** Relative mRNA expression of genes related to lipid absorption, lipid droplet formation and β-oxidation in neutrophils cocultured with HSCs from patients with CRLM (*n* = 4). **G** Representative images and relative cell viabilities of colorectal cancer patient-derived organoids cocultured with teHDNs and/or the human hepatic stellate cell line LX2 or LX2-CM (*n* = 3). **H** Lipid levels in the teHDNs treated with LX2 or LX2-CM (*n* = 3). **I**–**K** Lipid levels, TG contents and relative viability of the teHDNs treated with LX2-CM or tumor-activated LX2-CM (*n* = 3)

The liver is one of the main target organs for colorectal cancer metastasis and is also an organ with highly active lipid metabolism [3]. To investigate whether the liver affects the lipid metabolism of neutrophils during liver metastasis, we measured the lipid content in M-LDNs in the livers of tumor-bearing mice and found that it was significantly greater than that in M-LDNs in the blood (Fig. 1F). In addition, when the lipid droplets (LDs) in M-LDNs in mouse models of different stages of colorectal cancer were compared, we found that M-LDNs in metastatic liver tumors presented greater neutral lipid accumulation than did M-LDNs in tumor-free and nonmetastatic tumor-bearing mouse livers (Fig. 1G). This finding was confirmed by the triglyceride (TG) content in liver-infiltrating M-LDNs (Fig. 1H). As the tumor progressed, the proportion of recruited neutrophils in the liver increased; however, this increase had already occurred before metastasis (Fig. 1I). We compared the LDs in infiltrating neutrophils in primary and metastatic lesions from the same patient. Compared with those in normal liver tissues and primary lesions, neutrophils that infiltrated liver metastases presented greater lipid enrichment (Fig. 2A). Lipid staining of primary M-LDNs from humans and mice confirmed these results (Fig. 2B). Chemokines, especially CXCR2 ligands, play a role in recruiting neutrophils to various tumor microenvironments and affect the function of neutrophils [15, 19]. To identify which cells in the liver recruit M-LDNs and increase lipid accumulation, we compared the expression levels of CXCL2 in various cells in the livers of tumor-bearing and tumor-free mice. Only the hepatic stellate cells (HSCs) of tumor-bearing mice presented increased transcription levels of neutrophil chemokines (Fig. 2C). Immunostaining was performed on the livers of mice before metastasis, and the proximity of infiltrating neutrophils to HSCs indicated a potential impact of HSCs on M-LDNs (Fig. 2D). When neutrophils were cocultured with HSCs from tumor-bearing mice, we observed significant lipid accumulation in the teHDNs (Fig. 2E), and the CM of tumor-bearing HSCs also regulated the lipid metabolism of the M-LDNs (Fig. 2F). Organoids derived from patients with colorectal cancer were constructed and cocultured with teHDNs and/or the human hepatic stellate cell line LX2 to assess the effects of neutrophils and HSCs on tumor cells. The ability of teHDNs and LX2 to promote the proliferation of colorectal cancer organoids was observed to various degrees, with the most significant effect observed when both were present (Fig. 2G). However, LX2-CM did not exert the same effect as LX2 did (Fig. 2G). Further analyses of neutrophil lipid content in the coculture system revealed an increase in neutrophil lipids in the LX2 coculture group (Fig. 2H). Therefore, we speculate that when M-LDNs infiltrate the liver, they can be induced to accumulate lipids via proximal resident HSCs.

Only HSCs from tumor-bearing mice or LX2 cells cocultured with tumor cells, rather than LX2-CM, induced lipid accumulation in neutrophils. Therefore, we speculated that the changes in HSCs in the tumor environment led to this result. Through Sirius Red and Masson staining, we detected a significant amount of collagen deposition in liver metastases (Fig. S2A); notably, large quantities of collagen are produced when HSCs are activated. After treatment with CM from tumor cells, LX2 cells transformed from spindle shaped to radial, with increased protrusions (Fig. S2B), upregulated activation marker (ACTA2, COL1A1, DES) expression, and downregulated quiescence marker (RGS5, Fig. S2C) expression. Owing to the high expression of CXCL2/5 in the HSCs of tumor-bearing mice, we examined the expression levels of chemokines in tumor-activated LX2 (aLX2) cells. The expression of most chemokines was upregulated in activated HSCs (aHSCs, Fig. S2D). This enabled teHDNs to be recruited by aHSCs (Fig. S2E). Next, we examined whether HSC activation induced further lipid accumulation in neutrophils. As expected, aHSC-CM treatment and coculture had similar effects on the level of lipid accumulation (Fig. 2I), including the TG content (Fig. 2J), in neutrophils. A further comparison of neutrophil viability after different CM treatments was performed. Different CMs prolonged the survival of neutrophils by approximately 36 hours (Fig. 2K). Compared with HSC-CM, aHSC-CM had a greater ability to maintain neutrophil survival (Fig. 2K). As a result, metastatic liver-infiltrating M-LDNs induced by activated HSCs had greater neutral lipid accumulation than did blood M-LDNs.

### An increase in triglycerides in teHDNs is induced by activated hepatic stellate cells

**Fig. 3:**
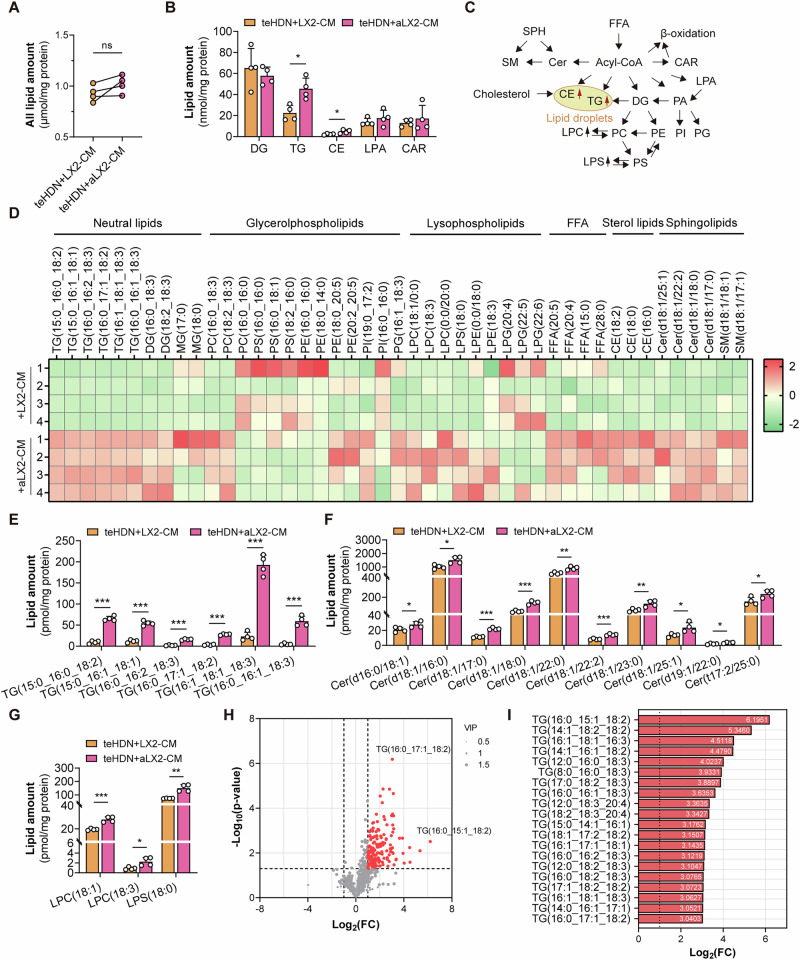
An increase in triglyceride synthesis in teHDNs was induced by activated hepatic stellate cells. **A** Total lipid content of teHDNs treated with LX2-CM or tumor-activated LX2-CM (*n* = 4). **B**, **E**–**G** Levels of representative individual lipid species in teHDNs treated with LX2-CM or tumor-activated LX2-CM (*n* = 4). DG, diglyceride. TG, triglyceride. CE, cholesteryl ester. LPA, lysophosphatidic acid. CAR, carnitine. Cer, ceramide. LPC, lysophosphatidylcholine. LPS, lysophosphatidylserine. **C** Summary of the transformation of different lipid species. **D** Heatmap of representative lipids in teHDNs treated with LX2-CM or tumor-activated LX2-CM analyzed via lipidomics (*n* = 4). MG, monoglyceride. PC, phosphatidylcholine. PS, phosphatidylserine. PE, phosphatidylethanolamine. PI, phosphatidylinositol. PG, phosphatidylglycerol. LPE, lysophosphatidylethanolamine. LPG, lysophosphatidylglycerol. FFA, free fatty acid. SM, sphingomyelin. **H** Volcano plots showing the fold changes and *P* values for the comparison of individual lipid species in teHDNs treated with LX2-CM or tumor-activated LX2-CM (*n* = 4). **I** The top 20 upregulated lipid species in teHDNs treated with tumor-activated LX2-CM (*n* = 4)

To analyze the changes in the classification of different lipids in neutrophils, we conducted lipidomic analysis. Surprisingly, compared with the control treatment, activated HSC treatment did not increase the overall lipid content in teHDNs but tended to increase it (Fig. 3A). Free fatty acids (FFAs) also tended to increase in the aHSC treatment group (Fig. S3A). Among the various lipids generated by FFAs, TGs and cholesterol esters (CEs) were significantly increased in the aHSC treatment group (Figs. 3B-E and  S3B). These two types of lipids are the main components of LDs. The content of carnitine (CAR) did not significantly change (Fig. 3B), indicating that lipids in teHDNs did not undergo β-oxidation after aHSC treatment. This finding was consistent with previous PCR results (Fig. S1A). Next, we extensively analyzed and compared the lipid distributions in the two groups. We found that ceramides (Cers) increased after stimulation with aHSC-CM (Fig. 3F). In addition, we found that in structural lipids, lysophosphatidylcholine (LPC) and lysophosphatidylserine (LPS) significantly increased, whereas phospholipids did not significantly change (Figs. 3G and S3C-G). The unsaturation of TGs and CEs increased to varying degrees (Fig. S3H). Furthermore, we conducted differential lipid analysis (*p* value < 0.05, FC > 2) and identified 154 increased lipids, of which 140 were TGs (Fig. 3H) and the top 20 were all TGs (Fig. 3I). However, no differentially decreased lipid species were found (Fig. 3H). Therefore, the lipid accumulation induced by activated HSCs in teHDNs is due mainly to an increase in the triglyceride content.

### DGAT-dependent lipid droplet formation maintains M-LDN survival and function

**Fig. 4:**
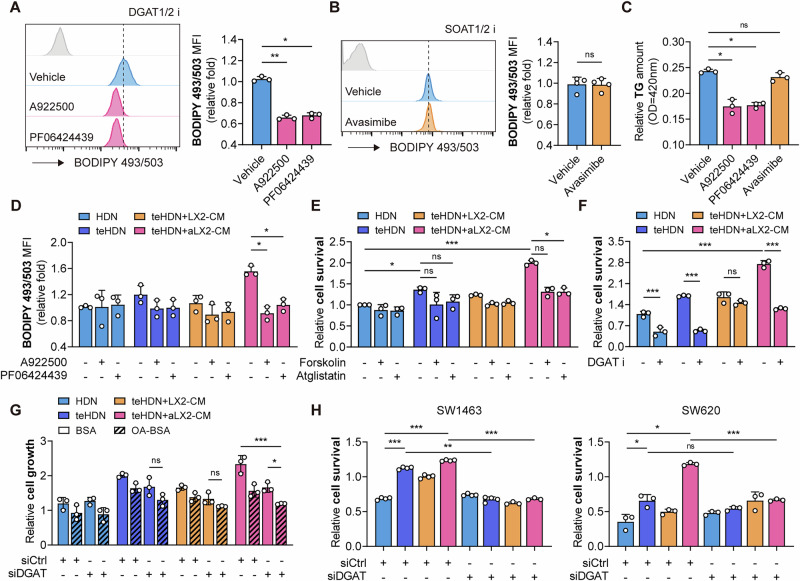
DGAT-dependent lipid droplet formation maintains M-LDN survival. **A**–**C** Lipid levels and relative TG contents in aLX2-CM-educated teHDNs treated with DGAT1/2 and SOAT1/2 inhibitors (A922500, PF06424439 and avasimibe, *n* = 3 or 4). **D** Lipid levels in DGAT1/2 inhibitor-treated HDNs, teHDNs, and teHDNs treated with LX2-CM or aLX2-CM (*n* = 3). **E** Relative survival of neutrophils treated with an adenylate cyclase activator (forskolin) or ATGL inhibitor (atglistatin) in low-serum medium (*n* = 3). **F** Relative survival of neutrophils with/without siRNA-mediated knockdown of DGAT1/2 (*n* = 3). **G** Cell growth extent of neutrophils following the transfection of DGAT1/2-targeting (or control) siRNA and treatment with 50 µM BSA-conjugated oleic acid (OA) for 72 hours (*n* = 3). **H** Relative survival of colorectal cancer cell lines (SW1463 and SW620) treated with the designated neutrophils (*n* = 4 or 3)

To investigate the source of TGs in teHDNs, we first examined whether the increase in TGs was due to an increase in lipid content in the culture supernatant of aLX2 cells. The results revealed that there was no significant change in the TG or FFA content in aLX2 CM (Fig. S4A). These findings indicate that activated HSCs do not increase the lipid content by directly delivering lipids or lipid raw materials to teHDNs. We then examined the uptake of fatty acids into M-HDNs. CD36, the main fatty acid transferase, is a key factor in mediating LD biogenesis through the uptake of exogenous FFAs [20–22]. The expression of CD36 in M-LDNs increased, especially in tumor tissues (Fig. S4B). We found that the LD content in M-LDNs did not decrease when the neutrophils were treated with the CD36 inhibitor sulfosuccinimidyl oleate (SSO) to inhibit FFA uptake (Fig. S4C). Therefore, we speculate that inhibiting the uptake of FFAs cannot reduce neutral lipid accumulation in M-LDNs, an effect that is due to the short period of in vitro inhibition, while subsequent lipid synthesis is still ongoing. That is, neutral lipid accumulation in M-LDNs is mainly the result of sustained lipid synthesis. To test this hypothesis, we examined the role of key enzymes involved in TG and CE synthesis in neutral lipid accumulation in M-LDNs. SOAT1/2 did not play a major role in M-LDN LD formation, whereas the pharmacological inhibition of DGAT1 and DGAT2 reduced neutral lipid accumulation and TG content and inhibited cell viability (Fig. 4A-C, Fig. S4D-E). This inhibitory effect was more pronounced in aHSC-stimulated teHDNs (Fig. 4D). The phenomenon we observed was different from the findings of previous reports that tumor cells rely solely on DGAT1 rather than DGAT2 to regulate TG synthesis and LD formation [23]. This difference may be due to the different expression patterns of DGAT1 and DGAT2 in neutrophils (Fig. S4F). In addition, compared with the inhibition of DGAT1 or DGAT2 alone, the simultaneous inhibition of DGAT1 and DGAT2 did not further reduce lipid droplet levels (Fig. S4G), potentially because DGAT1 and DGAT2 do not function simultaneously in neutrophils. In summary, DGAT1/2 is a key factor that mediates LD biogenesis through triglyceride synthesis. This explains why the expression of FFA transporters increased (Fig. S1A), but there was no significant increase in the level of intracellular FFAs (Fig. S3A).

LD formation is believed to prevent excessive intracellular FFAs from causing lipid toxicity to cells [24]. Destructive bioactive lipids are generated, and the integrity of mitochondrial membranes is disrupted when cells take up excessive external lipids or mobilized FFAs. We treated neutrophils with the adenylate cyclase activator forskolin to force lipid hydrolysis and acute LD clearance. We observed that cell death was accelerated by LD deprivation (Figs. 4E, and S4H). In addition, to inhibit the release of fatty acids from LDs, we blocked the activity of adipose triglyceride lipase (ATGL) via atglistatin and found that this treatment similarly accelerated the death of aLX2-CM-cultured teHDNs in low-serum medium (Figs. 4E and S4H). The upregulation of DGAT1/2 expression enables teHDNs to cope with the presence of excessive external lipids. In fact, the pharmacological inhibition of DGAT1/2 caused significant lipotoxicity in aLX2-CM-treated teHDNs, reducing cell survival and proliferation (Figs. 4F and S4I-K). To prevent compensatory upregulation of DGAT2 expression after knocking down DGAT1, or vice versa, we simultaneously knocked down DGAT1 and DGAT2 (Fig. S4L). OA is a potent inducer of TG synthesis, and excessive OA accumulation in cells can affect cell viability [25]. As expected, the addition of OA increased the accumulation of neutral lipids in teHDNs; after DGAT1/2 was knocked down, the accumulation of neutral lipids decreased (Fig. S4M). OA exposure preferentially suppressed the growth of DGAT1/2-inhibiting neutrophils (Fig. 4G). We examined the effect of DGAT1/2 inhibition on the function of teHDNs. Activated HSCs promoted the expression of the mature marker CD66b in neutrophils (Fig. S4N), reduced the ability of teHDNs to kill tumor cells and even promoted tumor cell growth (Fig. 4H). The inhibition of DGAT1/2 restored the killing effect of HDNs on tumor cells (Fig. 4H). Therefore, DGAT1/2-dependent LD formation in neutrophils maintains their survival and function.

Mitochondria are important targets of lipotoxicity [26]. We examined the changes in mitochondrial function in teHDNs after aLX2-CM treatment. The results revealed that aHSC-CM significantly increased the mitochondrial membrane potential of teHDNs (Fig. S5A-B) and reduced mitochondrial superoxide levels (Fig. S5C). To determine whether mitochondrial dysfunction is the main cause of teHDN cell death after DGAT1/2 inhibition, we assessed the morphology of teHDNs via transmission electron microscopy (TEM). Microscopy images revealed that after the inhibition of DGAT1/2, the mitochondrial structure was disrupted. Compared with the mitochondria in control cells, the mitochondria became fragmented and lost their cristae in cells in which DGAT1/2 was inhibited (Fig. S5D). In addition, the inhibition of DGAT1/2 significantly reduced the mitochondrial membrane potential (Fig. S5E-G) and increased the mitochondrial superoxide level (Fig. S5H). We found that treating cells with the ROS scavenger Mito-TEMPO reduced DGAT1/2 inhibition-induced cell death (Fig. S5I-J). Our data strongly suggest that DGAT1/2 is necessary for teHDNs/M-LDNs to cope with increases in lipid levels and maintain their survival and function. The mitochondrial damage caused by DGAT1/2 inhibition is the cause of teHDN death.

### IL33 secreted by aHSCs promotes lipid accumulation in M-LDNs

**Fig. 5:**
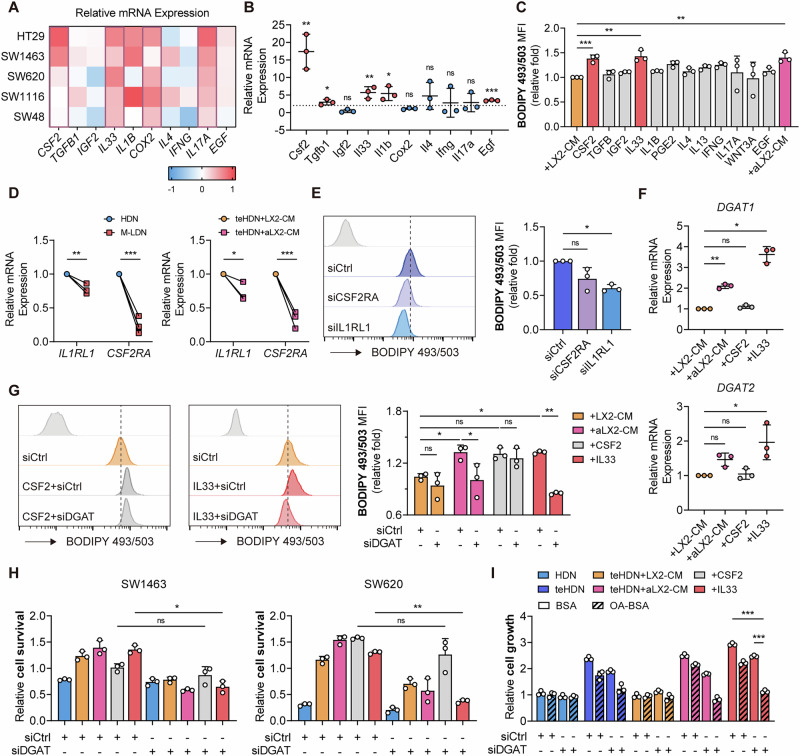
IL33 secreted by aHSCs promotes lipid accumulation in M-LDNs. **A** Relative mRNA expression of cytokines (CSF2, TGFB1, IGF2, IL33, IL1B, COX2, IL4, IFNG, IL17A, and EGF) in tumor cells treated with LX2. **B** Relative mRNA expression of cytokines in the HSCs of tumor-bearing and tumor-free mice (*n* = 3). **C** Lipid levels in teHDNs treated with recombinant cytokines (*n* = 3). **D** Relative mRNA expression of IL1RL1 and CSF2RA in HDNs, M-LDNs, and teHDNs treated with LX2-CM/aLX2-CM (*n* = 3). **E** Lipid levels in teHDNs with/without siRNA-mediated knockdown of CSF2RA or IL2RL1 (*n* = 3). **F** Relative mRNA expression of DGAT1 and DGAT2 in teHDNs treated with LX2-CM/aLX2-CM (*n* = 3). **G** Lipid levels in teHDNs with/without siRNA-mediated knockdown of DGAT1/2 treated with CSF2 or IL33 (*n* = 3). **H** Relative survival of SW1463 and SW620 cells cocultured with teHDNs with/without siRNA-mediated knockdown of DGAT1/2 (*n* = 3). **I** Cell growth extent of neutrophils with/without siRNA-mediated knockdown of DGAT1/2 and treatment with 50 µM BSA-conjugated oleic acid (OA) for 72 hours (*n* = 3)

Since both activated LX2 CM and LX2 CM can regulate lipid metabolism in teHDNs (Fig. 2H-I), we speculated that HSCs mainly regulate lipid accumulation in neutrophils by secreting soluble factors. By screening differentially expressed cytokines in LX2 cells activated by various tumor cells, we found that the expression of CSF2, IL33, COX2, and IL17A was upregulated (Fig. 5A). The differentially expressed cytokines between tumor-bearing and tumor-free mice included CSF2, TGFB1, IL33, IL1B, and EGF (Fig. 5B). After the HDNs were treated with these cytokines, CSF2 and IL33 were found to increase neutrophil lipid levels to a greater extent than other factors were (Fig. 5C). Interestingly, the expression of the IL33 receptor IL1RL1 was not upregulated in M-LDNs or aLX2-CM-treated teHDNs, whereas the expression of the CSF2 receptor CSF2RA was significantly downregulated (Figs. 5D, and  S6A). Knocking down the IL33 receptor but not the CSF2 receptor in teHDNs inhibited lipid accumulation in teHDNs (Fig. 5E). The expression of DGAT1/2 was upregulated after treatment with IL33 but not CSF2 (Figs. 5F and S6B). The increase in teHDN LD and TG levels induced by IL33 but not CSF2 was reduced by DGAT1/2 inhibition (Figs. 5G and S6C). Similarly, IL33 impaired the killing ability of HDNs against tumor cells, an effect that was rescued by DGAT1/2 inhibition; CSF2 did not impair the killing ability of HDNs against tumor cells (Fig. 5H). After the addition of OA, DGAT1/2-inhibited teHDNs treated with IL33 showed growth restriction (Fig. 5I). Therefore, IL33, derived from activated HSCs, is a potential cytokine that can regulate lipid accumulation in neutrophils.

CSF2 (also known as GM-CSF) is a crucial cytokine that promotes the proliferation, differentiation, and functional activation of myeloid cells, including neutrophils and macrophages [27]. To investigate whether HSCs enhance M-LDN lipid accumulation through synergistic effects or alternative pathways, we examined the regulatory effects of CSF2 on M-LDN lipids. Our analysis revealed that CSF2 treatment significantly upregulated genes associated with lipid uptake while downregulating those involved in fatty acid oxidation (Fig. S6D). Flow cytometry confirmed CSF2-induced CD36 expression (Fig. S6E). However, functional inhibition of CD36 did not attenuate CSF2-induced lipid accumulation in neutrophils (Fig. S6F), suggesting a minimal role of CD36 in M-LDN lipid deposition. Similarly, the knockdown of MSR1 and SCARB1 failed to significantly alter CSF2-mediated lipid accumulation (Fig. S6G). Further investigation into fatty acid oxidation in CSF2-treated teHDNs demonstrated that CSF2 suppresses this metabolic pathway (Fig. S6H). Notably, the activation of fatty acid oxidation via the AMPK agonist AICAR partially reversed CSF2-driven lipid accumulation (Fig. S6I-J). Fatty acid oxidation is a process further downstream of LD degradation, while lipolysis directly regulates the content of LDs. We investigated the effect of CSF2 on LD lipolysis in M-LDNs. After CSF2 treatment, the phosphorylation of HSL (a key lipolytic enzyme) and PLIN1 (a regulatory protein for lipolytic enzymes) decreased synchronously in teHDNs (Fig. S6K). The recruitment of ATGL (another key lipolytic enzyme) to lipid droplets was reduced (Fig. S6L), whereas the transcription levels of its main inhibitors, HILPDA and G0S2, increased (Fig. S6D). When a cAMP activator was added to stimulate lipolysis, the effect of CSF2 on increasing lipid accumulation was diminished (Fig. S6M). These findings collectively demonstrate that CSF2 primarily regulates teHDN lipid accumulation by modulating lipolysis and fatty acid oxidation.

### mTOR/PPARγ-regulated M-LDN lipid synthesis promotes cell proliferation and maintains immunosuppressive functions

**Fig. 6:**
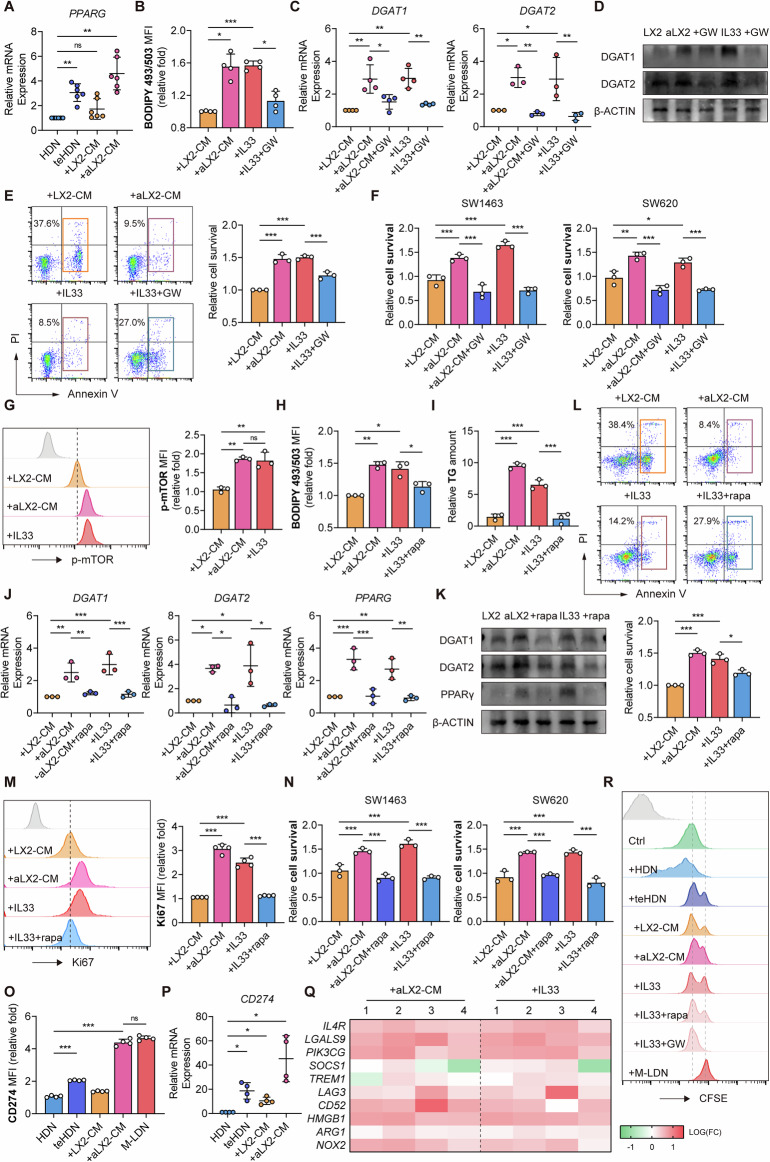
mTOR/PPARγ-regulated M-LDN lipid synthesis promotes cell proliferation and maintains immunosuppressive function. **A** Relative mRNA expression of PPARG in teHDNs treated with LX2-CM/aLX2-CM (*n* = 3). **B** Lipid levels in teHDNs treated with the PPARγ inhibitor GW9662 (*n* = 3). **C** Relative mRNA expression of DGAT1 and DGAT2 in designated teHDNs treated with GW9662 (*n* = 3). **D** Immunoblots of DGAT1 and DGAT2 in designated teHDNs treated with GW9662. **E** Relative survival of teHDNs treated with GW9662 (*n* = 3). **F** Relative survival of SW1463 and SW620 cells cocultured with teHDNs with/without GW9662 (*n* = 3). **G** Relative p-mTOR expression in teHDNs treated with LX2-CM/aLX2-CM or IL33 (*n* = 3). **H**, **I** Lipid levels and TG contents in teHDNs treated with the mTOR inhibitor rapamycin (*n* = 3). **J** Relative mRNA expression of DGAT1, DGAT2 and PPARG in designated teHDNs treated with rapamycin (*n* = 3). **K** Immunoblots of DGAT1, DGAT2 and PPARG in designated teHDNs treated with rapamycin. **L**, **M** Relative cell survival and Ki67 expression of teHDNs treated with rapamycin (*n* = 3). **N** Relative survival of SW1463 and SW620 cells cocultured with teHDNs with or without rapamycin (*n* = 3). **O**, **P** Relative CD274 expression in teHDNs treated with LX2-CM/aLX2-CM detected by flow cytometry (*n* = 4) and qPCR (*n* = 3). **Q** Relative mRNA expression of immune suppression-related genes in teHDNs treated with LX2-CM/aLX2-CM or IL33 (*n* = 4). **R** T-cell proliferation when cocultured with designated neutrophils

To better understand the regulation of TG metabolism in teHDNs, we investigated potential transcriptional regulatory factors. PPARγ, an important regulatory factor in lipid metabolism in adipose tissue and the liver, regulates FA uptake and LD formation [28]. Treatment with aLX2-CM resulted in the upregulation of PPARγ in teHDNs (Figs. 6A and S7A). To investigate the role of PPARγ in IL33-induced lipid accumulation in teHDNs, we cultured teHDNs with the specific PPARγ inhibitor GW9662. We found that the functional inhibition of PPARγ reduced lipid accumulation by decreasing the expression of DGAT1/2 (Figs. 6B-C and  S7B). The survival ability, proliferation ability, and ability to promote tumor cell proliferation of teHDNs were also inhibited (Figs. 6D-F, and S7C). Previous studies have suggested that mTOR is a major regulator of intracellular energy status-related signaling pathways and that mTOR may regulate lipid metabolism by modulating PPARγ [29, 30]. Treatment with aLX2-CM or IL33 increased the level of phosphorylated mTOR (Fig. 6G), which decreased after IL1RL1 was knocked down (Fig. S7D). The addition of the mTOR inhibitor rapamycin reduced IL33-induced LD formation and DGAT1/2 and PPARG expression, as well as the survival ability, proliferation ability, and ability to promote tumor cell proliferation of teHDNs (Fig. 6H-N). To validate the role of mTOR in IL33-mediated LD formation, we treated neutrophils with anti-IL33 neutralizing antibodies. Anti-IL33 neutralizing antibodies effectively reduced neutral lipid accumulation induced by aLX2-CM, decreased the expression of DGAT1/2 and PPARG, decreased the phosphorylation level of mTOR, and decreased the survival ability of teHDNs (Fig. S7E-I). In summary, these data indicate that lipid accumulation in teHDNs induced by IL33 is mediated by mTOR, which controls the expression of PPARγ and DGAT1/2.

To further investigate the intermediate signaling molecules mediating IL33-induced mTOR activation, we examined the expression levels of potential signal transduction proteins in teHDNs after IL33 treatment. The PI3K/AKT pathway is a canonical signaling cascade downstream of IL33/IL1RL1 activation [31]. Our results revealed that IL33 treatment increased AKT phosphorylation in teHDNs (Fig. S7J). Treatment with the PI3K inhibitor LY294002 or the AKT inhibitor MK-2206 attenuated the IL33-induced upregulation of pmTOR, PPARγ and DGATs (Fig. S7K). These results demonstrate that IL33 activates mTOR through PI3K/AKT signaling, leading to the transcriptional activation of downstream PPARγ/DGATs.

Notably, the inhibitory effect of neutrophils on CD8^+^ T-cell function is associated with tumor growth [16, 32]. Because our previous research indicated that M-LDNs mediate immunosuppressive functions and that activated HSCs can induce a significant increase in CD274 expression (Fig. 6O-P), we investigated whether lipid accumulation in M-LDNs affects this function. Compared with the control, stimulation with aLX2-CM or IL33 upregulated the expression of immune suppression-related genes, an effect that was reversed after treatment with an anti-IL33 neutralizing antibody (Figs. 6Q, and S7L). A coculture experiment with T cells also revealed that teHDNs treated with aLX2-CM or IL33 inhibited T-cell proliferation, an effect that was partially alleviated by mTOR inhibitors or PPARγ inhibitors (Fig. 6R). To exclude the direct effect of IL33 on tumor cells, we treated tumor cells with recombinant IL33. According to our results, IL33 did not directly increase the protein expression of PD-L1 or CD274 in tumor cells (Fig. S7M-N). Similarly, IL33 did not affect tumor cell proliferation (Fig. S7O). Therefore, IL33 mediates its immunosuppressive function by affecting mTOR/PPARγ-regulated LD formation in neutrophils.

Owing to the observation of active glucose metabolism in the teHDNs and M-LDNs (Fig. S1F), we speculated that the presence of glucose affects the function of neutrophils. To test this hypothesis, we evaluated the glucose uptake capacity of teHDNs. Treatment with aLX2-CM or IL33 increased glucose uptake by teHDNs, and treatment with mTOR inhibitors or PPARγ inhibitors decreased glucose uptake by teHDNs (Fig. S8A). The culture conditions without glucose reduced the ability of teHDNs to store obtained lipids in LDs; decreased their proliferation, DGAT1 expression, and mTOR phosphorylation levels; and decreased their survival ability and ability to promote tumor cell proliferation (Fig. S8B-G). These findings indicate that glucose availability may control neutral lipid accumulation by regulating the expression of DGAT1. We then investigated whether reducing glucose availability has a combined effect on the inhibition of DGAT1/2 production. The results revealed that reduced glucose levels decreased LD formation after DGAT1/2 inhibition (Fig. S8H-I). Moreover, the transcription level of CD274 decreased when DGAT1/2 was knocked down, and the inhibitory effect of teHDNs on T-cell proliferation was restored (Fig. S8J-K). In summary, the availability of glucose combined with IL33 promotes LD formation and the immunosuppressive function of teHDNs.

### Lipid-laden M-LDNs awaken tumor cells from dormancy and promote liver metastasis

**Fig. 7:**
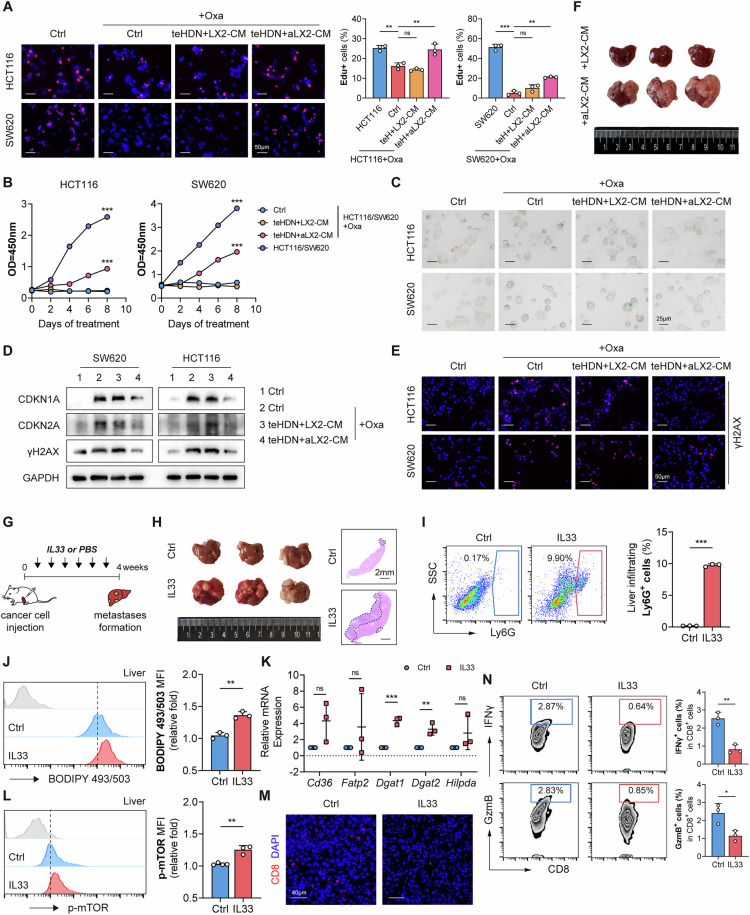
Lipid-laden M-LDNs awaken tumor cells from dormancy and promote liver metastasis. **A** Representative fluorescence images and relative percentages of EdU+ cells among neutrophil-CM-treated dormant HCT116 and SW620 cells (*n* = 3). Scale bar, 50 μm. **B** Growth curves of dormant HCT116 and SW620 cells treated with neutrophil CM (*n* = 3). **C** Representative images of senescence-associated β-galactosidase staining in neutrophil-CM-treated dormant HCT116 and SW620 cells (*n* = 5 images in total). Scale bar, 25 μm. **D** Representative immunoblotting of CDKN1A, CDKN2A and γH2AX in neutrophil-CM-treated dormant HCT116 and SW620 cells. **E** Representative fluorescence images of γH2AX in neutrophil-CM-treated dormant HCT116 and SW620 cells. **F** Photos of the livers of mice after intrasplenic transplantation with neutrophil-CM-treated dormant CT26 cells (*n* = 3). **G** Therapeutic scheme for IL33 treatment in BALB/c mice with liver metastasis after intrasplenic transplantation with CT26. Tumors were harvested and photographed at the end of the experiments. **H** Photos and H&E staining of livers from mice after IL33 treatment (*n* = 3). Scale bar (right), 2 mm. **I** Proportions of liver-infiltrating Ly6G^+^ neutrophils in the livers of IL33-treated mice (*n* = 3). **J** Lipid levels in liver-infiltrating M-LDNs after IL33 treatment (*n* = 3). **K** Relative mRNA expression of lipid metabolism genes in liver-infiltrating M-LDNs after IL33 treatment (*n* = 3). (**L**) Relative p-mTOR expression in liver-infiltrating M-LDNs after IL33 treatment (*n* = 3). **M** Representative fluorescence images of CD8 + T cells in IL33-treated mouse liver metastases (*n* = 5 images in total). Scale bar, 40 μm. **N** Proportions of liver-infiltrating IFNγ^+^ and GzmB^+^ CD8^+^ T cells in the livers of IL33-treated mice (*n* = 3)

Whether DTCs can colonize and grow into visible metastases in the liver depends on, to some extent, the dormant state of DTCs in the liver PMN [6]. We investigated whether lipid-laden neutrophils can promote liver metastasis by altering the dormant state of tumor cells. Transient treatment of tumor cells with high-dose DNA-damaging drugs (e.g., oxaliplatin) can induce dormancy, i.e., therapy-induced senescence [33, 34]. After coculturing dormant tumor cells with neutrophils, the proportion of proliferating cells among HCT116 and SW620 cells significantly increased with aLX2-CM treatment (Fig. 7A), and the viability of tumor cells also significantly increased after coculturing for approximately one week (Fig. 7B). Next, we measured the activity of a canonical marker of cell senescence, senescence-associated β-galactosidase (SA-β-gal). After aLX2-CM treatment, the increase in the percentage of SA-β-gal-positive cells was suppressed (Fig. 7C). Owing to the important role of the DNA damage response in inducing cellular aging, we next investigated the expression of the typical DNA damage marker γH2AX and the activity of the aging-related p53/p21 pathway mediated by the cellular DNA damage response after aLX2-CM treatment and observed the suppression of related molecules whose expression was previously upregulated (Fig. 7D-E). The ability of dormant tumor cells to form liver metastases after treatment with neutrophils was tested, and it was found that tumor cells treated with LX2-CM had difficulty forming lesions, whereas tumor cells treated with aLX2-CM regained this ability (Fig. 7F). Therefore, lipid-rich M-LDNs can reactivate dormant tumor cells and promote tumor growth in vitro.

To investigate whether this reactivation is mediated by secreted factors from lipid-rich neutrophils, we fractionated the CM on the basis of a molecular weight cutoff of 30 kDa, considering that most cytokines are under this threshold. The findings revealed that the >30 kDa CM fraction promoted the proliferation of dormant tumor cells (Fig. S9A-B) while reducing the expression of cell senescence markers (Fig. S9C), decreasing the expression of DNA damage markers, and downregulating the expression of senescence-associated p53/p21 pathway proteins (Fig. S9D-E). In contrast, the <30 kDa fraction failed to reactivate colorectal cancer cells. These results suggest that tumor cell reactivation is unlikely to be mediated through cytokine secretion by lipid-laden neutrophils.

We next cocultured dormant tumor cells with lipid-fluorescently labeled neutrophils or treated them with neutrophil CM. During the investigation of whether lipid-laden teHDNs could transfer lipids to tumor cells, we observed divergent outcomes with these approaches. Treatment with lipid-laden neutrophil-CM resulted in fluorescent signal detection within tumor cells, with dormant tumor cells exhibiting enhanced lipid acquisition capacity (Fig. S9F). These findings indicate that tumor cells exit dormancy by incorporating neutral lipids from lipid-laden neutrophils. However, when 0.4 μm Transwell coculture systems were used, no neutrophil-derived lipid transfer to tumor cells was detected (Fig. S9G). We therefore conclude that lipid transfer between neutrophils and tumor cells likely occurs through microvesicle or lipid droplet budding rather than via exosomes or hydrolyzed fatty acid transport. Furthermore, treatment of dormant tumor cells with extracted neutrophil lipid components resulted in increased proliferation rates (Fig. S10A), increased proliferative capacity (Fig. S10B), and decreased expression of DNA damage markers along with senescence-associated p53/p21 pathway proteins (Fig. S10C-D), confirming the functional role of neutrophil lipids in reactivation of dormancy in tumor cells.

To elucidate the alterations in lipid metabolism in dormant intestinal cancer cells under the influence of neutrophil-derived lipids, we performed lipidomic analysis. Interestingly, although dormant colorectal cancer cells took up lipid components from neutrophils, their overall lipid content, including TG, CE, PE, PS, and SPH, decreased (Fig. S10G-I). Moreover, the levels of FFAs, Cer, eicosanoids, and lysophospholipids significantly increased after neutrophil lipid treatment (Fig. S10G-I). These findings suggest that dormant tumor cells shift toward lipolytic catabolism upon lipid uptake (Fig. S11A). The elevated transcriptional levels of lipases—primarily ATGL, HSL and MGL (Fig. S11B)—confirmed accelerated TG degradation. Concurrently, we observed downregulation of lipid synthesis-related proteins (Fig. S11B), likely due to feedback inhibition from increased FFAs. Furthermore, after treatment, dormant tumor cells presented increased β-oxidation (Fig. S11C), elevated ATP levels (Fig. S11D), and upregulated CPT1A expression (Fig. S11B), collectively indicating that increased energy demand serves as the primary driver of their reactivation from dormancy.

Moreover, we observed a significant increase in eicosanoid levels (Fig. S10H), indicating that dormant tumor cells preferentially utilize FFAs for eicosanoid biosynthesis in addition to fueling β-oxidation for energy production. Eicosanoids are a group of bioactive lipid mediators derived from 20-carbon polyunsaturated fatty acids (e.g., arachidonic acids, AAs) through metabolic pathways mediated by cyclooxygenases (COXs), lipoxygenases (LOXs), and cytochrome P450 enzymes [35]. These compounds include prostaglandins (PGs), thromboxanes (TXs), leukotrienes (LTs), and lipoxins (LXs). Within the tumor microenvironment, eicosanoids significantly promote tumor progression by modulating inflammation, immune evasion, angiogenesis, and metastasis [36–38]. Elevated levels of intracellular FFAs, particularly saturated fatty acids (Fig. S11E), can activate cytosolic phospholipase A2 (cPLA2). Activated cPLA2 specifically hydrolyzes membrane phospholipids (e.g., phosphatidylinositol [PI] and phosphatidylethanolamine [PE]) to release AAs. Our results revealed a significant reduction in the levels of PS, PE, and major AA-containing membrane phospholipids (Fig. S11F), whereas the levels of the hydrolysis products lysophospholipids (LPI and LPS) markedly increased (Fig. S11G). These findings suggest that enhanced membrane phospholipid hydrolysis with insufficient reacylation repair leads to lysophospholipid accumulation and membrane phospholipid depletion. The released AAs are subsequently metabolized by COX and LOX enzymes to generate eicosanoids. We observed upregulated transcriptional levels of COX-1/COX-2 and 5-LOX/12-LOX/15-LOX (Fig. S11H), which directly contributed to elevated eicosanoid production. Notably, when a COX-2 inhibitor (NS-398) or LOX inhibitor (MK-886) was added to the neutrophil–lipid treatment system, the reactivation capacity of dormant tumor cells was significantly attenuated (Fig. S11I). Therefore, dormant colorectal cancer cells employ a dual metabolic strategy upon uptake of neutrophil-derived neutral lipids: increasing fatty acid oxidation to meet energy demands for reactivation and increasing membrane phospholipid hydrolysis to increase eicosanoid production, thereby activating the pro-proliferative pathway of tumor cells.

To better understand the role of HSC activation in vivo, we induced liver fibrosis via CCl_4_ [39] and constructed a colorectal cancer liver metastasis model in mice (Fig. S12A). The use of CCl_4_ increased the degree of liver fibrosis in the mice and significantly increased the occurrence of liver metastasis (Fig. S12B-C). Neutral lipid accumulation in M-LDNs in the peripheral blood of mice was increased in the CCl_4_ group, but the LD content in HDNs did not increase (Fig. S12D-E). The number of liver-infiltrating neutrophils significantly increased (Fig. S12F). The LD content increased (Fig. S12G), the expression of lipid metabolism-related genes increased (Fig. S12H), and mTOR phosphorylation levels increased (Fig. S12I) in liver-infiltrating M-LDNs. Immunofluorescence staining of the LD-associated protein Plin2 confirmed an increase in the lipid content of infiltrating neutrophils in the mouse liver (Fig. S12J). We isolated primary HSCs from mouse livers and detected an increase in the IL33 transcript level in the CCl_4_ group (Fig. S12K). We also observed significantly elevated IL33 levels in HSCs from mice at the concurrent liver metastasis stage (Fig. S12L). Moreover, we found that activated HSCs reduced the infiltration of CD8^+^ T cells in the mouse liver (Fig. S12J). Further analysis of the killing function of liver-infiltrating CD8^+^ T cells revealed a significant decrease in the expression levels of IFNγ and Granzyme B in T cells (Fig. S12M). Therefore, the activation of HSCs promotes lipid accumulation in M-LDNs, creates a fibrotic adipose microenvironment, and promotes the liver metastasis of colorectal cancer.

We used recombinant IL33 protein to validate the prometastatic effect of IL33 in vivo (Fig. 7G). As expected, IL33 promoted liver metastasis in mice with colorectal cancer (Fig. 7H). Concurrently, IL33 treatment increased neutrophil infiltration in the liver (Fig. 7I) and increased lipid accumulation, lipid metabolism-related gene expression, and mTOR phosphorylation in liver-infiltrating M-LDNs (Fig. 7J-L). The number of liver-infiltrating CD8^+^ T cells decreased after IL33 treatment (Fig. 7M), and the killing ability of CD8^+^ T cells decreased (Fig. 7N). These results indicate that IL33 plays a significant role in inducing M-LDN lipid accumulation and promoting liver metastasis.

### Inhibiting M-LDN lipid synthesis reduces liver metastasis and improves immunotherapy efficacy

**Fig. 8:**
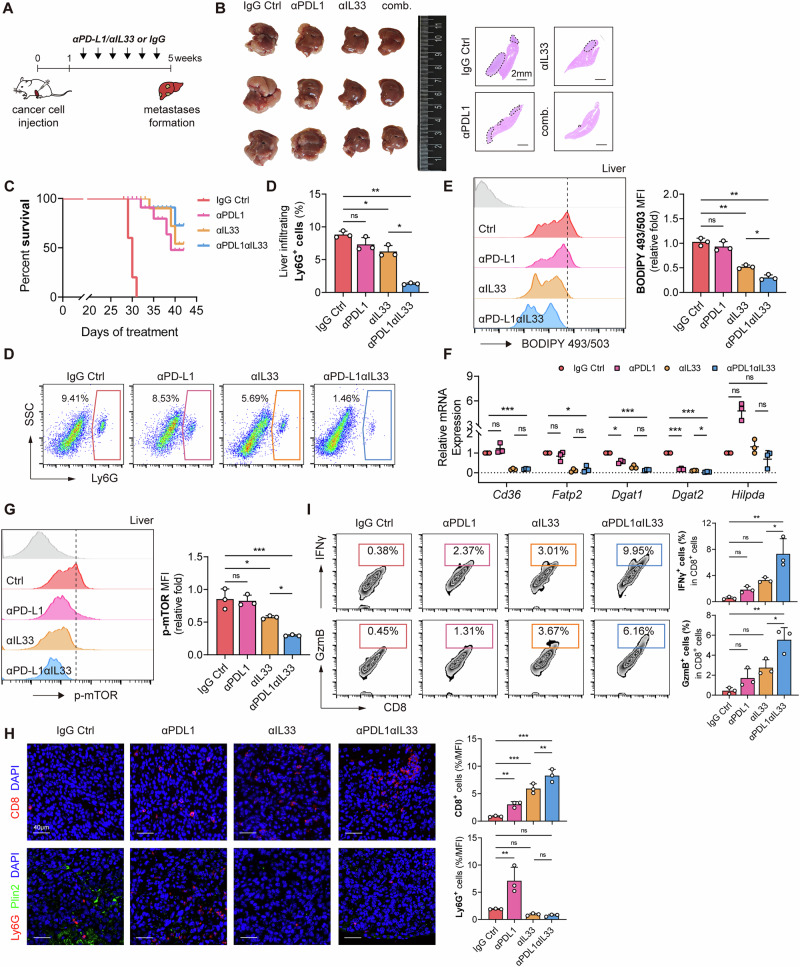
Inhibiting M-LDN lipid synthesis reduces liver metastasis and improves immunotherapy efficacy. **A** Therapeutic scheme for anti-IL33 and anti-PD-L1 treatment in BALB/c mice with liver metastasis after intrasplenic transplantation with CT26. Tumors were harvested and photographed at the end of the experiments. **B** Photos and H&E staining of livers from mice after anti-IL33 and anti-PD-L1 treatments (*n* = 3). Scale bar (right), 2 mm. **C** Survival curves of mice following anti-IL33 and anti-PD-L1 treatment. **D** Proportions of liver-infiltrating Ly6G^+^ neutrophils after anti-IL33 and anti-PD-L1 treatments (*n* = 3). **E** Lipid levels in liver-infiltrating M-LDNs after anti-IL33 and anti-PD-L1 treatments (*n* = 3). **F** Relative mRNA expression of lipid metabolism genes in liver-infiltrating M-LDNs after anti-IL33 and anti-PD-L1 treatments (*n* = 3). **G** Relative p-mTOR expression in liver-infiltrating M-LDNs after anti-IL33 and anti-PD-L1 treatments (*n* = 3). **H** Representative fluorescence images of Ly6G, Plin2 and CD8 in mouse liver metastases (*n* = 5 images in total). **I** Proportions of liver-infiltrating IFNγ^+^ and GzmB^+^ CD8^+^ T cells after anti-IL33 and anti-PD-L1 treatments (*n* = 3)

Owing to the immunosuppressive microenvironment, immune tolerance, and possible metabolic effects, the efficacy of immunotherapy in treating liver metastasis is limited [18]. Our previous results suggest that liver-infiltrating neutrophils have immunosuppressive effects. Thus, we analyzed the combined effect of M-LDN LD inhibition and immune checkpoint inhibitors (Fig. 8A). Both an anti-PD-L1 antibody and an anti-IL33 neutralizing antibody reduced the occurrence of liver metastasis, and this effect was more significant when both were used in combination (Fig. 8B). Combined anti-IL33 and anti-PD-L1 neutralizing antibody treatment prolonged survival in mice with liver metastases (Fig. 8C). Moreover, after treatment with the anti-IL33 neutralizing antibody, the proportion, lipid content, lipid metabolism-related gene expression and mTOR phosphorylation levels of liver-infiltrating M-LDNs decreased, especially when the anti-PD-L1 antibody and anti-IL33 neutralizing antibody were used in combination (Fig. 8D-G). Further analysis of liver-infiltrating CD8^+^ T cells revealed that when the anti-PD-L1 antibody and anti-IL33 neutralizing antibody were used in combination, the recruitment of CD8^+^ T cells to the liver increased, and the secretion of IFNγ and Granzyme B by CD8^+^ T cells increased (Fig. 8H-I). The essentially normal liver and kidney function parameters (e.g., ALT, AST, BUN, and Cr) in mice following combination therapy suggest that this regimen is a safe therapeutic approach (Fig. S13A-D). These results indicate that the inhibition of M-LDN lipid accumulation can reduce the occurrence of liver metastasis and increase the efficacy of immune checkpoint inhibitors.

Overall, our work revealed the crosstalk between HSCs and M-LDNs in the liver microenvironment and established the role of lipid metabolism in colorectal cancer liver metastasis. Activated HSCs induce lipid accumulation in M-LDNs, a process that is mediated by the IL33/mTOR/PPARγ/DGAT pathway. Lipid-laden M-LDNs promote metastatic colonization by transferring lipids to dormant tumor cells, thereby increasing lipid oxidation and eicosanoid biosynthesis, ultimately leading to the occurrence of colorectal cancer liver metastasis. Blocking LD formation in M-LDNs induced by IL33 inhibits liver metastasis and enhances the efficacy of immunotherapy.

## Discussion

The role of lipid metabolism changes in the biology of various solid tumors has attracted much attention, but how the lipid metabolism of neutrophils in the metastatic microenvironment affects the occurrence of metastasis has rarely been studied. In this study, we found that the extensive formation of neutrophil LDs maintains neutrophil survival and function and is an important factor in promoting DTC colonization in liver and liver metastases in patients with colorectal cancer. HSC activation initiates neutral lipid accumulation in liver-infiltrating M-LDNs.

HSC activation is considered a key factor affecting tumor progression in various tumors [40–42]. During the development of intrahepatic cholangiocarcinoma, activated HSCs transition into myofibroblasts (myCAFs) and inflammatory CAFs (iCAFs), which promote the progression of intrahepatic cholangiocarcinoma through the Has2/HA pathway and HGF/MET pathway, respectively [43, 44]. In addition, activated HSCs can deplete NK cells that maintain the dormancy of breast cancer cells, leading to liver metastasis [42]. Our results showed that lipid accumulation through TG synthesis rather than direct exogenous FFA uptake by M-LDNs in the premetastatic liver was regulated by HSC activation. This explains why neutrophils in a normal liver environment that is rich in lipids do not accumulate large amounts of LDs. Although HSCs exhibit considerable heterogeneity, established cell lines (including LX2) remain the predominant in vitro models for liver fibrosis research [40, 45, 46]. However, these cell lines lack critical functional characteristics of primary HSCs, thereby limiting their ability to mirror the heterogeneity observed in vivo. Primary human HSCs, while biologically heterogeneous, present substantial practical challenges: they are difficult to isolate, exhibit poor proliferative capacity, and cannot maintain quiescence under standard culture conditions. Consequently, novel experimental models that better recapitulate the phenotypic and functional properties of primary HSCs are urgently needed. Recent advances have demonstrated that induced pluripotent stem cell (iPSC)-derived HSC-like cells closely resemble primary human HSCs at the transcriptional, activation, and functional levels [47]. This innovative approach may represent an optimized strategy for future HSC research in vitro.

The increased secretion of IL33 by activated HSCs is the main reason for neutral lipid accumulation in liver-infiltrating M-LDNs. We observed that the expression level of IL33 in mouse HSCs was closely associated with liver metastasis status. However, under current conditions, we are unable to obtain clinical data correlating IL33 levels in patients with metastatic outcomes or clinical prognoses. Further exploration is still needed to support translational claims. The increased secretion of IL33 in tumor-associated fibroblasts has been reported to enhance the immunosuppressive function of ILC2s, Tregs and myeloid cells and promote the growth of pancreatic cancer [48]. In the metastatic microenvironment, the upregulation of fibroblast-derived IL33 expression triggers type 2 inflammation; mediates the recruitment of eosinophils, neutrophils and inflammatory monocytes to the lung; and promotes breast cancer metastasis [49]. Given that the IL33 receptor IL1RL1 is expressed by various immune cell types, blockade of IL33 may lead to potential off-target effects. However, IL33 predominantly suppresses immune responses through multiple mechanisms—mediating type 2 immune reactions, generating reparative M2 macrophages, inducing IL2 secretion, and selectively promoting Treg cell expansion [50]. Therefore, despite the potential systemic effects of IL33 blockade on other immune cells, we consistently observed reduced lipid content in lipid-laden neutrophils, decreased liver metastasis burden, and increased CD8^+^ T-cell recruitment. These findings sufficiently establish IL33 as a potential therapeutic target for controlling colorectal cancer liver metastasis. Certainly, it is essential to dissect the precise and multifaceted mechanisms of IL33 downstream signaling within the colorectal cancer liver metastasis microenvironment. This information will contribute to the clinical application of IL33 blockade combined with immunotherapy for the treatment of metastatic diseases.

Glucose is the main energy source for neutrophils. However, the reduced availability of glucose in the tumor microenvironment leads to metabolic reprogramming of neutrophils. In our study, we found that the activation of the DGAT1/2-TG synthesis pathway in neutrophils led to an increase in LD synthesis, which was particularly evident in the lipid-rich liver microenvironment. Lipid transporters are expressed at increased levels in M-LDNs. However, the uptake of FFAs in the liver microenvironment did not play a critical role in LD synthesis in liver-infiltrating M-LDNs. This contrasts with the prevalent view that cells continuously take up high levels of FFAs from the environment to synthesize TGs for fuel storage or that rapidly dividing cells rely on external FFAs to synthesize complex lipids for cell membranes [51]. The possible reason for this discrepancy is that, compared with tumor cells, neutrophils do not have a high basic demand for FFAs and can maintain or even increase LD synthesis after inhibiting FFA uptake. The formation of LDs is the main nutrient buffering system for the efficient storage of high-energy fuels [52]. This process plays a role mainly in adipose tissue. The main components of LDs, TGs and CEs, are elevated in M-LDNs in the liver microenvironment, but the data suggest that the SOAT-CE pathway does not play a major role. DGAT1 and DGAT2, key enzymes in TG synthesis, are present in the human liver and adipose tissue [53–55]. We found that the expression levels of DGAT1 and DGAT2 in M-LDNs were comparable. It has been reported that when DGAT1 function decreases, DGAT2 offsets potential toxic effects by sustaining TG synthesis [23]. Therefore, inhibiting the formation of TGs and LDs to trigger lipid toxicity in these cells requires the simultaneous inhibition of DGAT1 and DGAT2 [56]. Owing to the short duration of DGAT1/2 inhibition in neutrophils in our in vitro experiments, we did not observe compensatory upregulated expression after DGAT1/2 inhibition. However, these findings provide reference value for clinical trials investigating DGAT1/2 inhibition and liver metastasis in patients.

Our data indicated that LD synthesis was regulated by PPARγ, as the selective reduction in PPARγ activity directly inhibited LD formation as well as the survival, immune suppression, and tumor-promoting functions of liver-infiltrating M-LDNs. This finding aligns with previous reports indicating that PPARγ plays a crucial role in T-cell activation and the proliferation and aggregation of ILC2s during airway inflammation by directly regulating genes that control external lipid uptake [57, 58]. Interestingly, we found that the expression of DGAT1 was also partially regulated by glucose availability, particularly through the nutritional sensor mTOR. One potential explanation for this cross-regulation is that mitochondria closely associated with LDs rely on glycolysis for the generation of ATP, which is produced through oxidative phosphorylation and used for TG synthesis [59].

There is evidence that organ environments rich in lipids contribute to the successful colonization of DTCs and that tumor cells primarily promote their proliferation via the uptake of lipids [60–62]. Lipid-laden CAFs provide lipids for mitochondrial oxidative phosphorylation in cancer cells through ABCA8a transporters, thereby promoting pancreatic tumor progression [22]. In our study, we discovered that tumor cells can take up neutral lipids from lipid-laden neutrophils to exit dormancy. Specifically, dormant colorectal cancer cells utilize acquired lipids to increase fatty acid oxidation for energy production and increase membrane phospholipid hydrolysis to increase eicosanoid levels, thereby promoting proliferation. However, the specific species associated with elevated eicosanoids require further identification, and the precise mechanisms through which eicosanoids activate dormant tumor cells need further elucidation. Nonsteroidal anti-inflammatory drugs (NSAIDs) can inhibit COXs to block eicosanoid synthesis. The clinical potential of NSAIDs, or dual COX/LOX inhibitors (e.g., licofelone), in combination with immunotherapy to reduce the risk of colorectal cancer liver metastasis warrants further investigation. Overall, our findings support a proposed model whereby neutral lipid accumulation in liver-infiltrating M-LDNs potentiates both their cellular survival and immunomodulatory capacity, whereas subsequent lipid uptake by dormant tumor cells drives metabolic reprogramming through coordinated fatty acid β-oxidation and eicosanoid production, culminating in facilitated hepatic metastatic outgrowth. The neutrophil-induced reactivation of dormant colorectal cells is mechanistically driven by enhanced IL33 production from activated HSCs, resulting in consequential activation of the mTOR/PPARγ/DGAT-TG synthesis pathway. Our findings contribute to understanding the multifaceted functions of neutrophils in metastasis by revealing the regulatory roles of neutrophil lipid metabolism and energy availability. Moreover, the results of this study help elucidate the regulatory effects of M-LDN lipid accumulation on DTC dormancy and reveal that the ability of organ-specific stromal cells to regulate metabolism is a novel and potential therapeutic target for preventing and treating solid tumor metastasis.

## Materials and methods

### Patient samples and clinical data

This research received approval from the Ruijin Hospital Ethics Committee (NO. 2020--315) and was conducted in accordance with the Declaration of Helsinki, with written informed consent obtained from all patients involved. Whole blood samples and surgically resected tumor samples were acquired from patients with colorectal cancer at the Shanghai Minimally Invasive Surgery Center, Ruijin Hospital (Shanghai, China). For peripheral blood samples, immune cells were isolated and purified according to the methods described below for subsequent experiments. The available samples were used for organoid preparation. The clinical data of patients, including tumor pathological grading, were obtained from the medical records system. None of the patients received any treatment prior to surgery, and patients with inflammatory diseases, autoimmune diseases, or infections were excluded. The clinical and pathological details of the patients are provided in Tables S1 and S2.

### High-density and low-density neutrophil isolation

For peripheral blood samples, anticoagulated blood was diluted by half with phosphate-buffered saline (PBS). For tissue samples, the tumor tissues were washed with PBS containing penicillin and streptomycin, minced, added to RPMI 1640 medium containing 1 mg/mL collagenase IV (Gibco) and 5 mU/mL DNase I (Sigma‒Aldrich), and digested at 37°C for 45 minutes with rotation. After digestion, the tissue suspension was filtered through a 100 μm filter to obtain a single-cell suspension.

The process involves the formation of a double gradient on laying an equal volume of Histopaque-1077 (Sigma‒Aldrich) over Histopaque-1119 (Sigma‒Aldrich). Anticoagulated whole blood or single-cell tissue suspensions were gently placed on top of the upper Histopaque-1077 medium. High-density layer cells (granulocytes) are found at the lower Histopaque-1077/1119 interface, whereas low-density layer cells (lymphocytes, low-density neutrophils and other mononuclear cells) are found at the upper plasma/Histopaque-1077 interface. Low-speed centrifugation during the washing steps was used to remove most of the extraneous platelets from the upper interface. Erythrocytes were eliminated via red blood cell lysis buffer. To enrich neutrophils in the low-density layer, the cells were incubated with a biotin-conjugated anti-CD66b antibody (BioLegend) for 30 minutes at 4°C and then labeled with magnetic microbeads (Miltenyi Biotec) for 15 minutes, followed by magnetic-activated cell sorting (MACS).

### Isolation of primary hepatic stellate cells

After perfusion with solutions containing collagenase IV (Gibco) and DNase I (Sigma‒Aldrich), the crushed liver was digested for another 25 minutes. The cell suspension was filtered through a 70 µm cell strainer and rinsed with Gey’s balanced salt solution (GBSS). The cells were subjected to gradient centrifugation with 9.7% Nycodenz (Serumwerk Bernburg AG) for the mouse samples and 18% for the human samples to isolate the HSCs, which were then cultured in DMEM.

### BODIPY 493/503, Oil Red O and 2-NBDG staining

For the measurement of the cellular lipid contents, the cells were resuspended in medium supplemented with 10 μM BODIPY 493/503 (Cayman) for 15 minutes at 37°C in the dark and washed twice for further flow cytometry detection or microscopic observation. Relative lipid droplet numbers were quantified via flow cytometry.

To identify the key pathway of lipid synthesis or screen candidate soluble factors, HDNs were stimulated with the candidate regulators for 18 h, and then, the lipid levels of the neutrophils were analyzed using BODIPY 493/503 staining and flow cytometry. The final concentrations of each reagent were as follows: CSF2, TGFβ, IGF2, IL33, IL1β, PGE2, IL4, IL13, IFNγ, IL17A, WNT3A, and EGF, 100 ng/ml; sulfosuccinimidyl oleate, 100 mM; avasimibe, 10 μM; A922500, PF06424439, 20 μM; GW9662, 40 μM; rapamycin, 20 nM; and anti-IL33 antibody, 0.2 μg/ml.

Oil Red O staining was performed according to the manufacturer’s protocol (Solarbio). Neutrophils were spun onto slides and fixed in formaldehyde-calcium fixative at room temperature for 10 minutes. After being rinsed fully in distilled water and then with 60% isopropanol for 5–10 s, the cells were stained with Oil Red O working solution for 20 minutes. Then, 60% isopropanol was used for differentiation for 10 seconds to clear the mesenchyme, Mayer’s hematoxylin solution was used to redye for 5 minutes, and tap water was used to return the color to blue. The slides were observed under a microscope. For glucose uptake detection, the cells were resuspended in medium supplemented with 20 μM 2-NBDG (Cayman) for 15 minutes at 37°C in the dark and washed twice for further flow cytometry.

### Triglyceride and free fatty acid content assay

The levels of total triglycerides (TGs) and free fatty acids (FFAs) in the serum and conditioned medium were measured with commercial kits following the manufacturer’s protocol (Solarbio) and were determined with a microplate reader with a multiwavelength measurement system (Tecan Infinite® 200 PRO).

### Lipidomic analysis

After isolation, the neutrophils were immediately frozen in liquid nitrogen and stored at -80°C. Lipid contents were detected via MetWare (http://www.metware.cn/). Briefly, the sample was thawed on ice. Lipids were extracted via the MTBE method, and lipidomic analysis was conducted via an LC‒ESI‒MS/MS system (UPLC, ExionLC AD; MS, QTRAP® 6500+ System). The total sample amount was standardized according to the protein concentration.

### Flow cytometry and apoptosis assay

Human neutrophils were washed with fluorescence-activated cell sorting (FACS) buffer after treatment and then stained with the following antibodies: anti-CD36 (336207, BioLegend), anti-Ki67 (151209, BioLegend), anti-CD66b (305106, BioLegend), anti-p-mTOR (12-9718-41, Invitrogen), or anti-CD274 (561787, BD Biosciences). For mouse liver tissues, single-cell suspensions were stained with Ly6G (560599, BD Biosciences), CD8a (553030, BD Biosciences), IFN-γ (562018, BD Biosciences) and/or GzmB (396413, BioLegend). After being stained for 30 minutes at 4°C in the dark and washed with FACS buffer, the samples were detected with a flow cytometer (BD Biosciences). All the data were analyzed with FlowJo version 10 (BD Biosciences).

For the apoptosis assay, after treatment with culture medium, drugs or the control, the neutrophils were labeled with Annexin-V-APC (BD Biosciences) and propidium iodide (PI; BD Biosciences) according to the manufacturer’s instructions and analyzed using a flow cytometer (BD Biosciences). Annexin-V+ PI- cells were classified as early apoptotic cells, and Annexin-V+ PI+ cells were classified as late apoptotic cells. To determine the function of lipid droplets, HDNs were treated with 50 μM forskolin or atglistatin for 18 hours and then subjected to an apoptosis assay.

### T-cell proliferation assay

T cells were purified from peripheral blood low-density layer cells via an anti-CD3 antibody (Miltenyi Biotec) and the MACS method. The cells were then resuspended at 1×10^7^ cells/mL in prewarmed PBS containing CSFE (1 µl/mL, Invitrogen) and stained for 20 minutes at room temperature with gentle agitation. Any dye remaining in the solution was then quenched via a brief wash with complete medium. The T cells were inoculated in RPMI 1640 medium containing 20 IU/mL IL-2 at a density of 2×10^6^ cells/mL and stimulated with anti-CD3/CD28 antibodies (IBA Life Sciences). The purified neutrophils were cocultured with T cells according to the experimental design. After 5 days of culture, the CFSE levels were detected via flow cytometry.

### Induction of diverse colon cancer cell types and cell coculture

To generate therapy-induced senescence, cancer cells were treated with high-dose DNA damaging drugs (100 μM oxaliplatin) for 24 hours. The cells were subsequently cultured in fresh culture medium. For coculture, neutrophils were added to the upper transwell chamber (0.4 μm for 24-well plates; Corning Costar), and cancer cells were added to the lower chamber. After coculture for 48 hours, the cancer cells were stained with EdU and β-gal or seeded in 96-well plates cultured with neutrophil CM for the cell viability assay. SA-β-gal staining was performed via a Senescence β-Galactosidase Staining Kit (Cell Signaling Technology) following the manufacturer’s instructions.

### Animal studies

All the animal experiments were conducted with male BALB/c mice aged 4–6 weeks. All experiments were performed following the official suggestions of the Chinese Zoological Society and were authorized by the Ruijin Hospital Ethics Committee (No. 2020--315). To establish the liver metastasis model, the mice were anesthetized with 200 μl/10 g 2.5% tribromoethyl alcohol (Sigma‒Aldrich). A left abdominal wall incision was made to expose the spleen, and 1×10^7^ cells suspended in 100 μl of PBS were slowly injected into the spleen. The spleen was subsequently replaced into the abdominal cavity, and the incision was closed. Four to five weeks after injection, the mice were euthanized and examined for liver metastasis. The number of metastatic nodules in the liver was counted.

For the liver fibrosis induction model, the mice were treated with 50 μl/10 g 10% CCl_4_ (in olive oil) twice a week for six weeks before the spleen was injected with the tumor cells. For drug therapy, the mice were intraperitoneally injected with 3.6 μg/mouse recombinant murine IL33 (R&D Systems; PBS control), 5 μg/mouse anti-IL33 antibody (AF3626, R&D Systems; AB-108-C, R&D Systems), or 200 μg/mouse anti-PD-L1 antibody (CP001, BioXCell; IgG control, BE0083, BioXCell) twice a week for four weeks.

### Statistical analysis

All the statistical tests were performed with GraphPad Prism 8 (San Diego, USA). The normality of the data was first tested via the Shapiro‒Wilk test. For data that conformed to a normal distribution, Student’s *t* test was used to compare the significance between the two groups. Otherwise, the Mann‒Whitney test was used for unpaired comparisons, and the Wilcoxon test was used for paired sample significance analysis. ANOVA with Tukey’s or Sidak’s multiple comparisons test was used for comparisons among multiple groups. *P* values < 0.05 were considered significant (*P < 0.05, **P < 0.01, and ***P < 0.001).

## Supplementary information

Supplementary materials

## Data Availability

All the data are available in the main text or the supplementary materials.

## References

[1] Bergers G, Fendt SM. The metabolism of cancer cells during metastasis. Nat Rev Cancer. 2021;21:162–80. 33462499 10.1038/s41568-020-00320-2 PMC8733955

[2] Zhou H, Liu Z, Wang Y, Wen X, Amador EH, Yuan L, et al. Colorectal liver metastasis: molecular mechanism and interventional therapy. Signal Transduct Target Ther. 2022;7:70. 35246503 10.1038/s41392-022-00922-2 PMC8897452

[3] Tsilimigras DI, Brodt P, Clavien PA, Muschel RJ, D’Angelica MI, Endo I, et al. Liver metastases. Nat Rev Dis Prim. 2021;7:27. 33859205 10.1038/s41572-021-00261-6

[4] Lyden D, Ghajar CM, Correia AL, Aguirre-Ghiso JA, Cai S, Rescigno M, et al. Metastasis. Cancer Cell. 2022;40:787–91. 35944497 10.1016/j.ccell.2022.07.010 PMC9924435

[5] Liu Y, Cao X. Characteristics and Significance of the Premetastatic Niche. Cancer Cell. 2016;30:668–81. 27846389 10.1016/j.ccell.2016.09.011

[6] Massagué J, Obenauf AC. Metastatic colonization by circulating tumor cells. Nature. 2016;529:298–306. 26791720 10.1038/nature17038 PMC5029466

[7] Gerstberger S, Jiang Q, Ganesh K. Metastasis. Cell. 2023;186:1564–79. 37059065 10.1016/j.cell.2023.03.003 PMC10511214

[8] Patras L, Shaashua L, Matei I, Lyden D. Immune determinants of the premetastatic niche. Cancer Cell. 2023;41:546–72. 36917952 10.1016/j.ccell.2023.02.018 PMC10170403

[9] Yofe I, Shami T, Cohen N, Landsberger T, Sheban F, Stoler-Barak L, et al. Spatial and Temporal Mapping of Breast Cancer Lung Metastases Identify TREM2 Macrophages as Regulators of the Metastatic Boundary. Cancer Discov. 2023;13:2610–31. 37756565 10.1158/2159-8290.CD-23-0299 PMC7617931

[10] Theivanthiran B, Yarla N, Haykal T, Nguyen YV, Cao L, Ferreira M, et al. Tumor-intrinsic NLRP3-HSP70-TLR4 axis drives premetastatic niche development and hyperprogression during anti-PD-1 immunotherapy. Sci Transl Med. 2022;14:eabq7019. 36417489 10.1126/scitranslmed.abq7019 PMC10347419

[11] Cho Y, Bukong TN, Tornai D, Babuta M, Vlachos IS, Kanata E, et al. Neutrophil extracellular traps contribute to liver damage and increase defective low-density neutrophils in alcohol-associated hepatitis. J Hepatol. 2023;78:28–44. 36063965 10.1016/j.jhep.2022.08.029 PMC11910133

[12] Ng MSF, Kwok I, Tan L, Shi C, Cerezo-Wallis D, Tan Y, et al. Deterministic reprogramming of neutrophils within tumors. Science. 2024;383:eadf6493. 38207030 10.1126/science.adf6493 PMC11087151

[13] Sadiku P, Willson JA, Ryan EM, Sammut D, Coelho P, Watts ER, et al. Neutrophils Fuel Effective Immune Responses through Gluconeogenesis and Glycogenesis. Cell Metab. 2021;33:411–23.e4. 33306983 10.1016/j.cmet.2020.11.016 PMC7863914

[14] Ancey PB, Contat C, Boivin G, Sabatino S, Pascual J, Zangger N, et al. GLUT1 Expression in Tumor-Associated Neutrophils Promotes Lung Cancer Growth and Resistance to Radiotherapy. Cancer Res. 2021;81:2345–57. 33753374 10.1158/0008-5472.CAN-20-2870 PMC8137580

[15] Xue Y, Chen Y, Sun S, Tong X, Chen Y, Tang S, et al. TET2-STAT3-CXCL5 nexus promotes neutrophil lipid transfer to fuel lung adeno-to-squamous transition. J Exp Med. 2024;221:e20240111. 10.1084/jem.20240111 PMC11129275 38805014

[16] Biswas SK. Metabolic Reprogramming of Immune Cells in Cancer Progression. Immunity. 2015;43:435–49. 26377897 10.1016/j.immuni.2015.09.001

[17] Veglia F, Tyurin VA, Blasi M, De Leo A, Kossenkov AV, Donthireddy L, et al. Fatty acid transport protein 2 reprograms neutrophils in cancer. Nature. 2019;569:73–8. 30996346 10.1038/s41586-019-1118-2 PMC6557120

[18] Yu J, Green MD, Li S, Sun Y, Journey SN, Choi JE, et al. Liver metastasis restrains immunotherapy efficacy via macrophage-mediated T-cell elimination. Nat Med. 2021;27:152–64. 33398162 10.1038/s41591-020-1131-x PMC8095049

[19] Kolaczkowska E, Kubes P. Neutrophil recruitment and function in health and inflammation. Nat Rev Immunol. 2013;13:159–75. 23435331 10.1038/nri3399

[20] Corbet C, Bastien E, Santiago de Jesus JP, Dierge E, Martherus R, Vander Linden C, et al. TGFβ2-induced formation of lipid droplets supports acidosis-driven EMT and the metastatic spreading of cancer cells. Nat Commun. 2020;11:454. 31974393 10.1038/s41467-019-14262-3 PMC6978517

[21] Zhu GQ, Tang Z, Huang R, Qu WF, Fang Y, Yang R, et al. CD36(+) cancer-associated fibroblasts provide immunosuppressive microenvironment for hepatocellular carcinoma via secretion of macrophage migration inhibitory factor. Cell Discov. 2023;9:25. 36878933 10.1038/s41421-023-00529-z PMC9988869

[22] Niu N, Shen X, Wang Z, Chen Y, Weng Y, Yu F, et al. Tumor cell-intrinsic epigenetic dysregulation shapes cancer-associated fibroblasts heterogeneity to metabolically support pancreatic cancer. Cancer Cell. 2024;42:869–84.e9. 38579725 10.1016/j.ccell.2024.03.005

[23] Cheng X, Geng F, Pan M, Wu X, Zhong Y, Wang C, et al. Targeting DGAT1 Ameliorates Glioblastoma by Increasing Fat Catabolism and Oxidative Stress. Cell Metab. 2020;32:229–42.e8. 32559414 10.1016/j.cmet.2020.06.002 PMC7415721

[24] Olzmann JA, Carvalho P. Dynamics and functions of lipid droplets. Nat Rev Mol Cell Biol. 2019;20:137–55. 30523332 10.1038/s41580-018-0085-z PMC6746329

[25] Bagchi S, Yuan R, Huang HL, Zhang W, Chiu DK, Kim H, et al. The acid-sensing receptor GPR65 on tumor macrophages drives tumor growth in obesity. Sci Immunol. 2024;9:eadg6453. 39423285 10.1126/sciimmunol.adg6453 PMC12104511

[26] Fromenty B, Roden M. Mitochondrial alterations in fatty liver diseases. J Hepatol. 2023;78:415–29. 36209983 10.1016/j.jhep.2022.09.020

[27] Rapoport AP, Abboud CN, DiPersio JF. Granulocyte-macrophage colony-stimulating factor (GM-CSF) and granulocyte colony-stimulating factor (G-CSF): receptor biology, signal transduction, and neutrophil activation. Blood Rev. 1992;6:43–57. 1375123 10.1016/0268-960x(92)90007-d

[28] Ahmadian M, Suh JM, Hah N, Liddle C, Atkins AR, Downes M, et al. PPARγ signaling and metabolism: the good, the bad and the future. Nat Med. 2013;19:557–66. 23652116 10.1038/nm.3159 PMC3870016

[29] Bennett CF, Latorre-Muro P, Puigserver P. Mechanisms of mitochondrial respiratory adaptation. Nat Rev Mol Cell Biol. 2022;23:817–35. 35804199 10.1038/s41580-022-00506-6 PMC9926497

[30] Mossmann D, Park S, Hall MN. mTOR signaling and cellular metabolism are mutual determinants in cancer. Nat Rev Cancer. 2018;18:744–57. 30425336 10.1038/s41568-018-0074-8

[31] He D, Xu H, Zhang H, Tang R, Lan Y, Xing R, et al. Disruption of the IL-33-ST2-AKT signaling axis impairs neurodevelopment by inhibiting microglial metabolic adaptation and phagocytic function. Immunity. 2022;55:159–73.e9. 34982959 10.1016/j.immuni.2021.12.001 PMC9074730

[32] Bianchi A, De Castro Silva I, Deshpande NU, Singh S, Mehra S, Garrido VT, et al. Cell-Autonomous Cxcl1 Sustains Tolerogenic Circuitries and Stromal Inflammation via Neutrophil-Derived TNF in Pancreatic Cancer. Cancer Discov. 2023;13:1428–53. 36946782 10.1158/2159-8290.CD-22-1046 PMC10259764

[33] Duro-Sánchez S, Nadal-Serrano M, Lalinde-Gutiérrez M, Arenas EJ, Bernadó Morales C, Morancho B, et al. Therapy-Induced Senescence Enhances the Efficacy of HER2-Targeted Antibody‒Drug Conjugates in Breast Cancer. Cancer Res. 2022;82:4670–9. 36222720 10.1158/0008-5472.CAN-22-0787 PMC9755966

[34] Fitsiou E, Soto-Gamez A, Demaria M. Biological functions of therapy-induced senescence in cancer. Semin Cancer Biol. 2022;81:5–13. 33775830 10.1016/j.semcancer.2021.03.021

[35] Wang D, Dubois RN. Eicosanoids and cancer. Nat Rev Cancer. 2010;10:181–93. 20168319 10.1038/nrc2809 PMC2898136

[36] Bayerl F, Meiser P, Donakonda S, Hirschberger A, Lacher SB, Pedde AM, et al. Tumor-derived prostaglandin E2 programs cDC1 dysfunction to impair intratumoral orchestration of anticancer T cell responses. Immunity. 2023;56:1341–58.e11. 37315536 10.1016/j.immuni.2023.05.011

[37] Yu L, Liebenberg K, Shen Y, Liu F, Xu Z, Hao X, et al. Tumor-derived arachidonic acid reprograms neutrophils to promote immune suppression and therapy resistance in triple-negative breast cancer. Immunity. 2025;58:909–25.e7. 40157359 10.1016/j.immuni.2025.03.002 PMC11981829

[38] Elewaut A, Estivill G, Bayerl F, Castillon L, Novatchkova M, Pottendorfer E, et al. Cancer cells impair monocyte-mediated T-cell stimulation to evade immunity. Nature. 2025;637:716–25. 39604727 10.1038/s41586-024-08257-4 PMC7617236

[39] Brown ZJ, Heinrich B, Greten TF. Mouse models of hepatocellular carcinoma: an overview and highlights for immunotherapy research. Nat Rev Gastroenterol Hepatol. 2018;15:536–54. 29904153 10.1038/s41575-018-0033-6

[40] Qi M, Fan S, Huang M, Pan J, Li Y, Miao Q, et al. Targeting FAPα-expressing hepatic stellate cells overcomes resistance to antiangiogenics in colorectal cancer liver metastasis models. J Clin Investig. 2022;132:e157399. 10.1172/JCI157399 PMC9525122 35951441

[41] Wang SS, Tang XT, Lin M, Yuan J, Peng YJ, Yin X, et al. Perivenous Stellate Cells Are the Main Source of Myofibroblasts and Cancer-Associated Fibroblasts Formed After Chronic Liver Injuries. Hepatology. 2021;74:1578–94. 33817801 10.1002/hep.31848

[42] Correia AL, Guimaraes JC, Auf der Maur P, De Silva D, Trefny MP, Okamoto R, et al. Hepatic stellate cells suppress NK cell-sustained breast cancer dormancy. Nature. 2021;594:566–71. 34079127 10.1038/s41586-021-03614-z

[43] Affo S, Nair A, Brundu F, Ravichandra A, Bhattacharjee S, Matsuda M, et al. Promotion of cholangiocarcinoma growth by diverse cancer-associated fibroblast subpopulations. Cancer Cell. 2021;39:866–82.e11. 33930309 10.1016/j.ccell.2021.03.012 PMC8241235

[44] Bhattacharjee S, Hamberger F, Ravichandra A, Miller M, Nair A, Affo S, et al. Tumor restriction by type I collagen opposes tumor-promoting effects of cancer-associated fibroblasts. J Clin Investig. 2021;131:e146987. 10.1172/JCI146987 PMC8159701 33905375

[45] Gan L, Jiang Q, Huang D, Wu X, Zhu X, Wang L, et al. A natural small molecule alleviates liver fibrosis by targeting apolipoprotein L2. Nat Chem Biol. 2025;21:80–90. 39103634 10.1038/s41589-024-01704-3

[46] Myojin Y, Hikita H, Sugiyama M, Sasaki Y, Fukumoto K, Sakane S, et al. Hepatic Stellate Cells in Hepatocellular Carcinoma Promote Tumor Growth Via Growth Differentiation Factor 15 Production. Gastroenterology. 2021;160:1741–54.e16. 33346004 10.1053/j.gastro.2020.12.015

[47] Coll M, Perea L, Boon R, Leite SB, Vallverdú J, Mannaerts I, et al. Generation of Hepatic Stellate Cells from Human Pluripotent Stem Cells Enables In Vitro Modeling of Liver Fibrosis. Cell Stem Cell. 2018;23:101–13.e7. 30049452 10.1016/j.stem.2018.05.027

[48] Donahue KL, Watkoske HR, Kadiyala P, Du W, Brown K, Scales MK, et al. Oncogenic KRAS-Dependent Stromal Interleukin-33 Directs the Pancreatic Microenvironment to Promote Tumor Growth. Cancer Discov. 2024;14:1964–89. 38958646 10.1158/2159-8290.CD-24-0100 PMC11450371

[49] Shani O, Vorobyov T, Monteran L, Lavie D, Cohen N, Raz Y, et al. Fibroblast-Derived IL33 Facilitates Breast Cancer Metastasis by Modifying the Immune Microenvironment and Driving Type 2 Immunity. Cancer Res. 2020;80:5317–29. 33023944 10.1158/0008-5472.CAN-20-2116 PMC7611300

[50] Liew FY, Girard JP, Turnquist HR. Interleukin-33 in health and disease. Nat Rev Immunol. 2016;16:676–89. 27640624 10.1038/nri.2016.95

[51] Yao CH, Fowle-Grider R, Mahieu NG, Liu GY, Chen YJ, Wang R, et al. Exogenous Fatty Acids Are the Preferred Source of Membrane Lipids in Proliferating Fibroblasts. Cell Chem Biol. 2016;23:483–93. 27049668 10.1016/j.chembiol.2016.03.007 PMC5510604

[52] Marcelin G, Chua S Jr. Contributions of adipocyte lipid metabolism to body fat content and implications for the treatment of obesity. Curr Opin Pharm. 2010;10:588–93. 10.1016/j.coph.2010.05.008 PMC2945394 20860920

[53] Cases S, Smith SJ, Zheng YW, Myers HM, Lear SR, Sande E, et al. Identification of a gene encoding an acyl CoA:diacylglycerol acyltransferase, a key enzyme in triacylglycerol synthesis. Proc Natl Acad Sci USA. 1998;95:13018–23. 9789033 10.1073/pnas.95.22.13018 PMC23692

[54] Cases S, Stone SJ, Zhou P, Yen E, Tow B, Lardizabal KD, et al. Cloning of DGAT2, a second mammalian diacylglycerol acyltransferase, and related family members. J Biol Chem. 2001;276:38870–6. 11481335 10.1074/jbc.M106219200

[55] Harris CA, Haas JT, Streeper RS, Stone SJ, Kumari M, Yang K, et al. DGAT enzymes are required for triacylglycerol synthesis and lipid droplets in adipocytes. J Lipid Res. 2011;52:657–67. 21317108 10.1194/jlr.M013003 PMC3284159

[56] Ackerman D, Tumanov S, Qiu B, Michalopoulou E, Spata M, Azzam A, et al. Triglycerides Promote Lipid Homeostasis during Hypoxic Stress by Balancing Fatty Acid Saturation. Cell Rep. 2018;24:2596–605.e5. 30184495 10.1016/j.celrep.2018.08.015 PMC6137821

[57] Angela M, Endo Y, Asou HK, Yamamoto T, Tumes DJ, Tokuyama H, et al. Fatty acid metabolic reprogramming via mTOR-mediated inductions of PPARγ directs early activation of T cells. Nat Commun. 2016;7:13683. 27901044 10.1038/ncomms13683 PMC5141517

[58] Karagiannis F, Masouleh SK, Wunderling K, Surendar J, Schmitt V, Kazakov A, et al. Lipid-Droplet Formation Drives Pathogenic Group 2 Innate Lymphoid Cells in Airway Inflammation. Immunity. 2020;52:620–34.e6. 32268121 10.1016/j.immuni.2020.03.003

[59] Benador IY, Veliova M, Mahdaviani K, Petcherski A, Wikstrom JD, Assali EA, et al. Mitochondria Bound to Lipid Droplets Have Unique Bioenergetics, Composition, and Dynamics that Support Lipid Droplet Expansion. Cell Metab. 2018;27:869–85.e6. 29617645 10.1016/j.cmet.2018.03.003 PMC5969538

[60] Nieman KM, Kenny HA, Penicka CV, Ladanyi A, Buell-Gutbrod R, Zillhardt MR, et al. Adipocytes promote ovarian cancer metastasis and provide energy for rapid tumor growth. Nat Med. 2011;17:1498–503. 22037646 10.1038/nm.2492 PMC4157349

[61] Li P, Lu M, Shi J, Gong Z, Hua L, Li Q, et al. Lung mesenchymal cells elicit lipid storage in neutrophils that fuel breast cancer lung metastasis. Nat Immunol. 2020;21:1444–55. 32958928 10.1038/s41590-020-0783-5 PMC7584447

[62] Pascual G, Avgustinova A, Mejetta S, Martín M, Castellanos A, Attolini CS, et al. Targeting metastasis-initiating cells through the fatty acid receptor CD36. Nature. 2017;541:41–5. 27974793 10.1038/nature20791

