# Supplementary material for: IL33-induced lipid droplet formation in mature low-density neutrophils drives colorectal cancer liver metastasis
Source: Cell Mol Immunol. 2025 Nov 10;22(12):1598–614. doi: 10.1038/s41423-025-01365-9 (PMC12660769; doi:10.1038/s41423-025-01365-9)
Supplement: Supplementary file 1 — Supplementary materials [file 41423_2025_1365_MOESM1_ESM.docx]

**Supplementary Materials**

**Supplementary Materials and Methods**

**Cell culture**

Human CRC cell lines SW1463, SW620 and HCT116 cells were purchased from the American Type Culture Collection (ATCC). Mouse CRC cell line CT26 was purchased from Shanghai Institutes for Biological Sciences, Chinese Academy of Sciences. All cells were cultured in RPMI 1640 medium (Meilunbio) supplemented with 10% fetal bovine serum (FBS; Gibco) and 1% penicillin/streptomycin (Gibco) in a humidified incubator at 37 ℃ in 5% CO_2_. Human hepatic stellate cell line LX2 were purchased from ATCC and was cultured in DMEM medium (Meilunbio).

**Conditioned medium (CM) preparation**

When the adherent cells reached 100% confluence, the cell culture medium was replaced with fresh medium. For the cell suspension, 1×10^7^ cells were cultured in 1ml fresh medium. The cell culture medium was collected after 24 hours.

**Cell viability assay**

The ATP levels which represent the viability of neutrophils were detected by CellTiter-Lumi™ Steady Luminescent Cell Viability Assay Kit (Beyotime) according to the manufacturer’s protocol. Briefly, 100μl CellTiter-Lumi™ Reagent was added to each well, and the plates were then vigorously shaken for 2 minutes. Luminescence was measured after 10 minutes incubation at room temperature.

Cell survival rates were estimated by the Cell Counting Kit-8 (CCK-8) assay (Beyotime). Approximately 5,000 normal cells or 10,000 oxaliplatin-treated dormant cells were seeded in 96-well plates. Dormant cancer cells were treated with neutrophil conditioned medium. The viabilities of cells were detected every two days. When detection, each well was incubated with 10μl CCK-8 solution for two hours at 37℃ in the dark, and the absorbance at 450nm was measured by a microplate spectrophotometer (Tecan).

**Transwell assay**

For the neutrophil chemotaxis experiment, HDNs or teHDNs were added to the upper transwell chamber (8μm for 24-well plates, Corning Costar), and LX2 or aLX2 were added to the lower chamber. After 48 hours’ coculture, the number of neutrophils in the lower chamber was counted.

**siRNA knockdown**

Cells were transiently transfected with specific siRNA (GenePharma, China) using Lipofectamine™ 2000 (Invitrogen) following the protocol provided by the manufacturer. The targeting site sequences were as follows: si-DGAT1: TTTACTACGTGCTCAACTATG; si-DGAT2: TTATAGACTATTTGCTTTCAA; si-IL1RL1: TTACACCGTGGATTGGTATTA; si-CSF2RA: TTCGTGCACATTTCGTGAAAT; si-MSR1: TTCTTTAGGACGAAAGAAGTA; si-SCARB1: TTGTGAATCTCATCAACAAGT.

**Reactive oxygen species (ROS) and mitochondrial membrane potential detection**

After washing cells twice times with PBS, neutrophils were placed into RPMI 1640 supplemented with 5μM MitoSOX Red (Abclonal) for 15 minutes at 37℃ for ROS detection or 1×JC-1 staining buffer (Beyotime) for 20 minutes at 37℃ for mitochondrial membrane potential detection. After washing twice with PBS, cells were detected by a flow cytometer.

**EdU cell proliferation assay**

Cancer cells were treated with BeyoClick™ EdU Cell Proliferation Kit with Alexa Fluor 555 (Beyotime) according to the manufacturer’s instructions. The fluorescence was observed using a fluorescence microscope and quantified by the ImageJ software.

**Histological analysis**

For paraffin-embedded sections, the tumor tissue slices were stained using a sirius red staining kit, and a masson trichrome kit (Servicebio) to detect the collagen following the manufacturer’s instructions.

**RNA extraction and quantitative real-time PCR (qRT-PCR)**

Total RNA was extracted from the purified neutrophils or HSCs using TRIzol reagent (Invitrogen) according to the manufacturer's instructions. The RNA concentration was measured by Nanodrop 2000 (Thermo Scientific) and was reverse transcribed to cDNA using HiScript III RT SuperMix (Vazyme). The qPCR was performed using SYBR Green (Vazyme) according to the manufacturer's instructions. The sequences of the primers for the qPCR were listed in Table S2. The relative mRNA expression was calculated using the 2^−ΔΔCT^ method.

**Fatty Acid Oxidation (FAO) Assay**

The neutrophil FAO activity was measured using a fatty acid oxidation assay kit according to the manufacturer's instructions. Neutrophil extracts were prepared using lysis buffer, and the protein concentration of the lysate was determined by BCA protein assay. The FAO assay solution and 20×FAO substrate were thawed as needed, and control and reaction solutions were prepared. Samples were loaded into a 96-well plate, followed by the addition of control or reaction solutions, and incubated at 37°C for 30 min. The absorbance at 492 nm was measured using a microplate reader. FAO activity was calculated based on the absorbance difference between the reaction and control wells, with sample FAO activity (IU/L) = ΔO.D. × 12.96, and normalized to protein concentration.

**Lipid Extraction**

Lipid extraction was performed following the kit's protocol. Briefly, neutrophils were washed and resuspended in PBS. After adding proprietary organic compounds, the mixture underwent gravity-based phase separation via centrifugation. Total intracellular lipids were extracted and isolated by transferring them to the chloroform-free upper organic phase, followed by overnight drying in a desiccating incubator for subsequent experiments.

**Supplementary Figures**


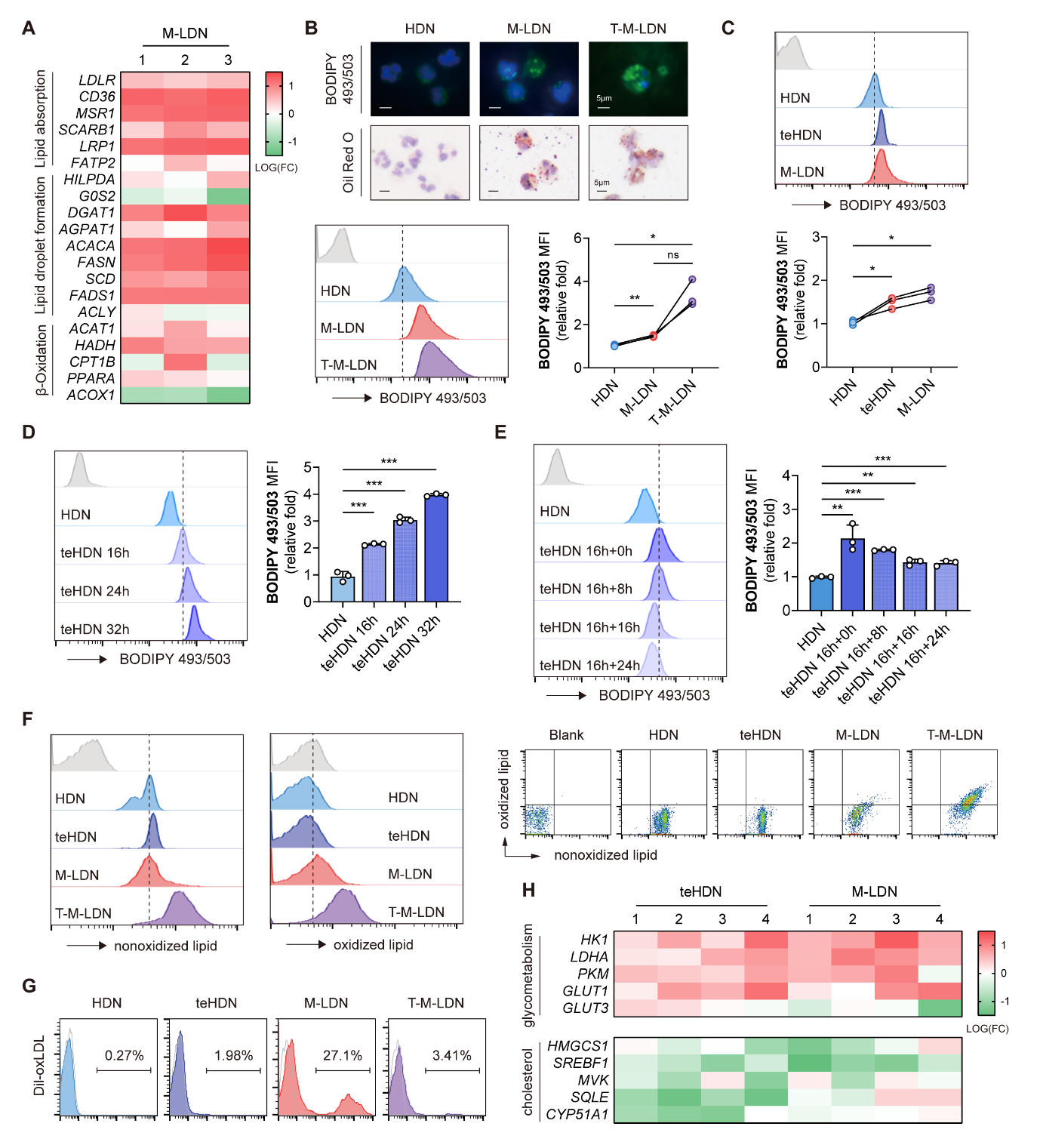


**Fig. S1 Colorectal cancer cells induce lipid accumulation in neutrophils and transition to low-density neutrophils.** (**A**) Relative mRNA expression of genes related to lipid absorption, lipid droplet formation and β-oxidation in M-LDNs (*n*=3). (**B**) Lipid staining of blood and tumor-infiltrating neutrophils in colorectal cancer patients detected by BODIPY 493/503 and Oil Red O. Lipid quantification by flow cytometry (*n*=3). Scale bar, 5μm. (**C**) Lipid levels in HDNs treated with tumor cell conditioned medium (teHDNs, *n*=3). (**D**) Lipid levels in HDNs treated with tumor CM for 16h, 24h and 32h (*n*=3). (**E**) Lipid levels in teHDNs post 8h, 16h and 24h withdrawal after tumor CM treatment (*n*=3). (**F-G**) Oxidized, nonoxidized lipid and Dil-oxLDL uptake levels of HDNs, teHDNs, M-LDNs and tumor-infiltrating M-LDNs (*n*=3). (**H**) Relative mRNA expression of genes related to glycometabolism and cholesterol metabolism in teHDNs and M-LDNs (*n*=4).


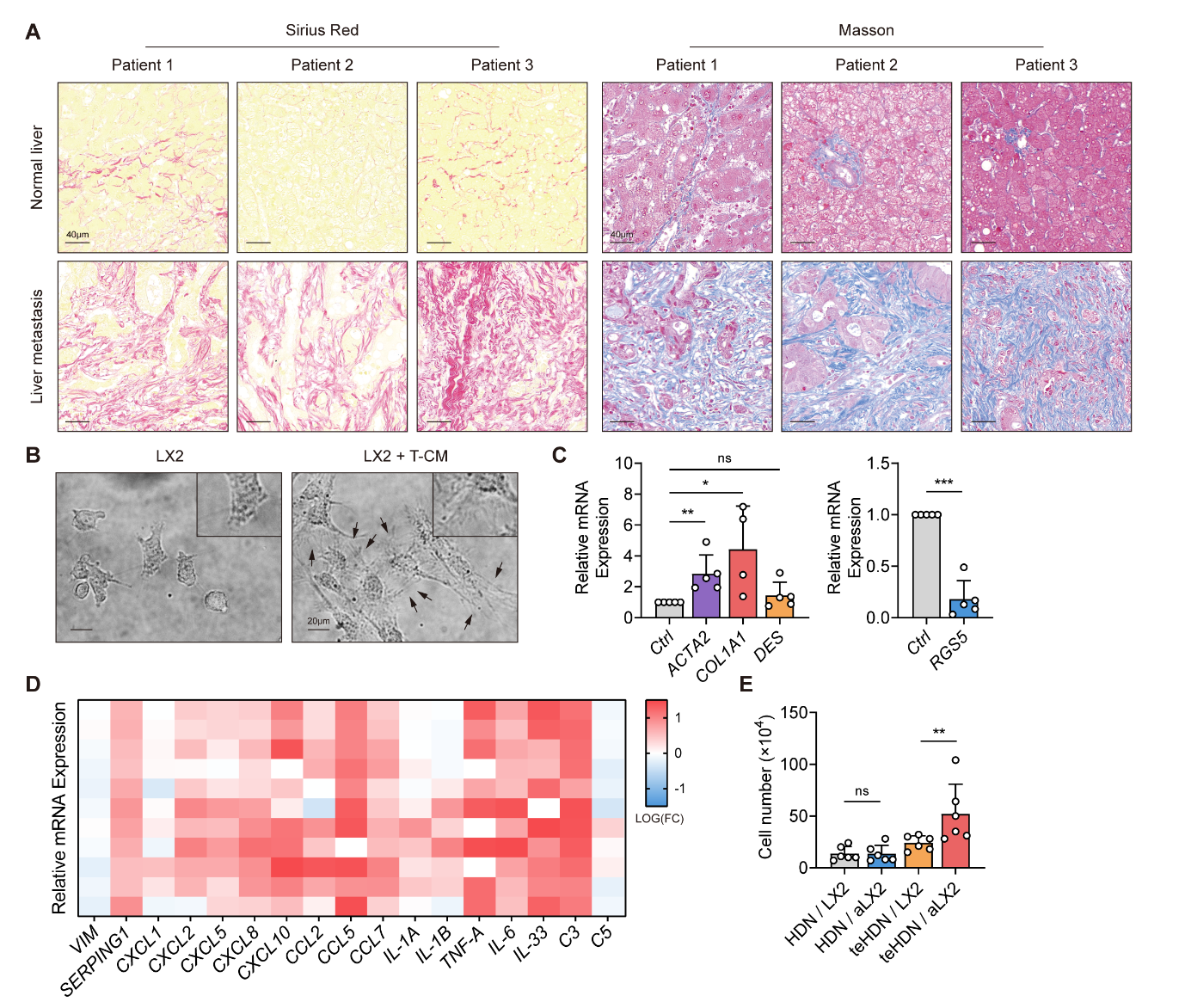


**Fig. S2 Hepatic stellate cells can be activated by colorectal cancer cells.** (**A**) The representative pictures of normal liver and liver metastasis tissues stained with Sirius Red and Masson (representative of *n*=5). Scale bar, 40μm. (**B**) The morphological images of LX2 treated with or without tumor conditioned medium. Scale bar, 20μm. (**C**) Relative mRNA expression of activation and quiescence markers of HSC treated with conditioned medium (*n*=4 or 5). (**D**) Relative mRNA expression of chemokines in tumor-activated LX2 (*n*=11). (**E**) The number of neutrophils that pass through in the transwell assay (*n*=6).


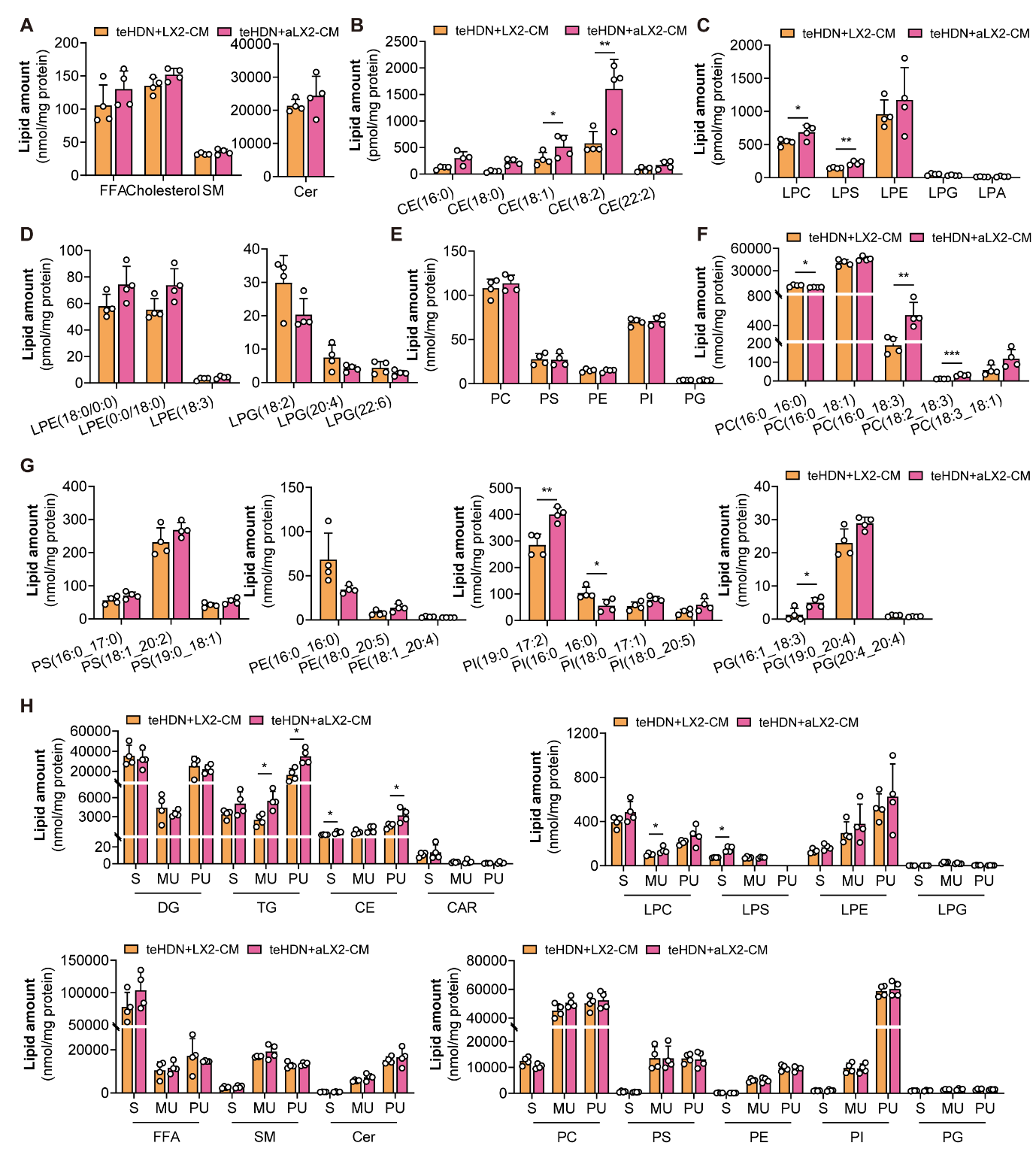


**Fig. S3 The content of different lipid classes in teHDNs treated with activated HSC-CM.** (**A-G**) Levels of representative individual lipid species in teHDNs treated with LX2-CM or tumor-activated LX2-CM (*n*=4). (**H**) Degree of unsaturation of representative individual lipid species in teHDNs treated with LX2-CM or tumor-activated LX2-CM (*n*=4). FFA, free fatty acid. SM, sphingomyelin. Cer, ceramide. CE, cholesteryl ester. LPC, lysophosphatidylcholine. LPS, lysophosphatidylserine. LPE, lysophosphatidylethanolamine. LPG, lysophosphatidylglycerol. LPA, lysophosphatidic acid. PC, phosphatidylcholine. PS, phosphatidylserine. PE, phosphatidylethanolamine. PI, phosphatidylinositol. PG, phosphatidylglycerol. DG, diglyceride. TG, triglyceride. CAR, carnitine. S, saturated. MU, monounsaturated. PU, polyunsaturated.


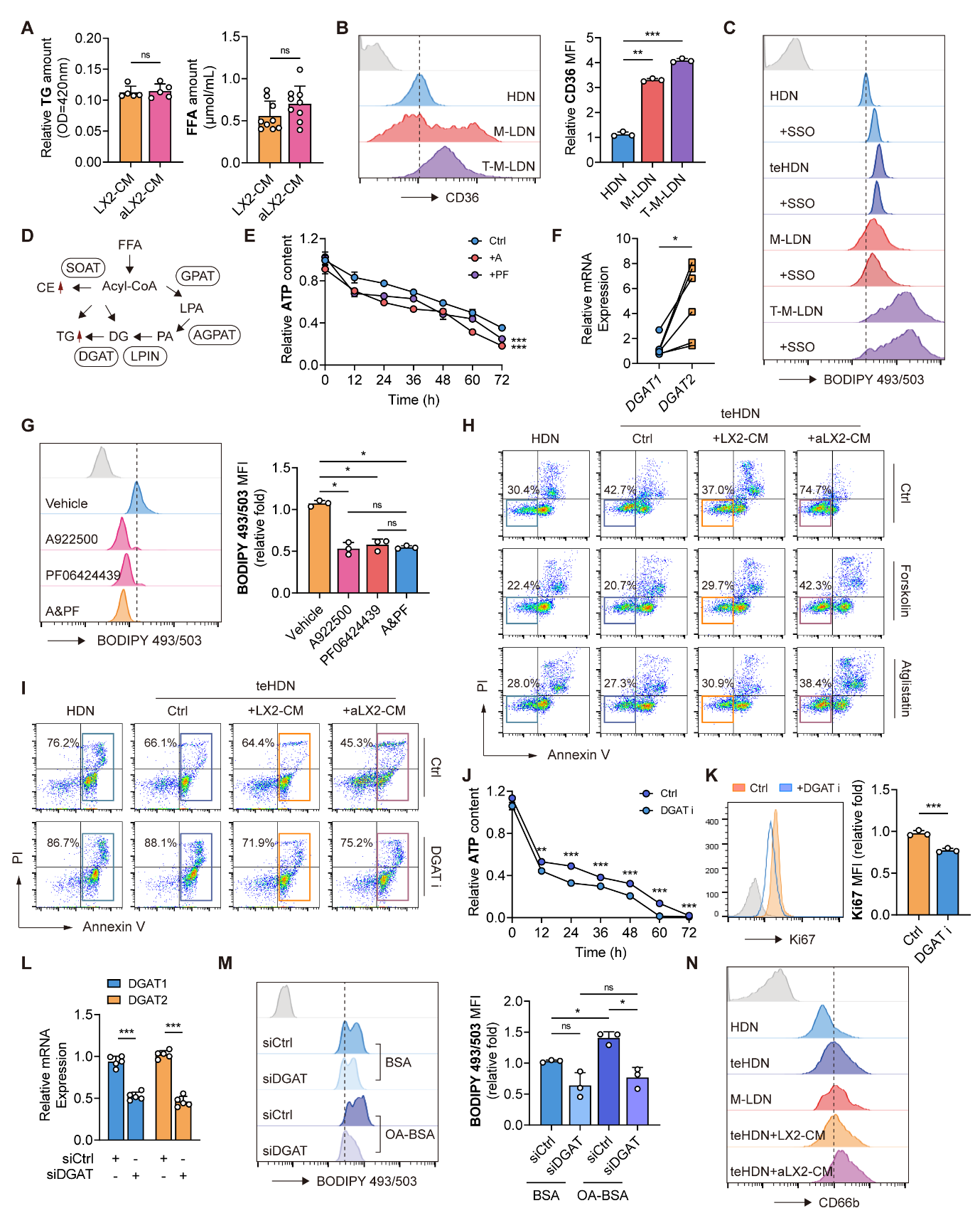


**Fig. S4 CD36-dependent lipid uptake does not participate in aHSC-driven increase in M-LDN lipid droplets.** (**A**) Relative TG amount and FFA amount of LX2-CM and aLX2-CM (*n*=5 or 9). (**B**) CD36 expression of HDNs, M-LDNs and tumor-infiltrating M-LDNs detected by flow cytometry (*n*=3). (**C**) Lipid levels in HDNs, teHDNs, M-LDNs and tumor-infiltrating M-LDNs treated with CD36 inhibitor sulfosuccinimidyl oleate (SSO, representative of *n*=3). (**D**) Summary of key enzymes in lipid synthesis. (**E**) Cell viability changes in aLX2-CM educated teHDNs treated with DGAT1 and DGAT2 inhibitors (A922500 and PF06424439, *n*=3). (**F**) Relative mRNA expression of DGAT1 and DGAT2 in neutrophils (*n*=6). (**G**) Lipid levels in teHDNs treated with DGAT1 and DGAT2 inhibitors (*n*=3). (**H**) Apoptosis assay of neutrophils treated with adenylate cyclase activator (forskolin) and ATGL inhibitor (atglistatin) in low serum medium (*n*=3). (**I**) Apoptosis assay of neutrophils treated with DGAT1/2 inhibitors (*n*=3). (**J-K**) Cell viability changes and Ki67 expression in aLX2-CM educated teHDNs treated with DGAT1/2 inhibitors (*n*=3). (**L**) Relative mRNA expression of DGAT1 and DGAT2 with/without siRNA knockdown of DGAT1/2 (*n*=5). (**M**) Lipid levels in BSA or OA-BSA treated neutrophils with/without siRNA knockdown of DGAT1/2 (*n*=3). (**N**) CD66b expression of HDNs, teHDNs, M-LDNs and teHDNs treated with LX2-CM or aLX2-CM (representative of *n*=3).


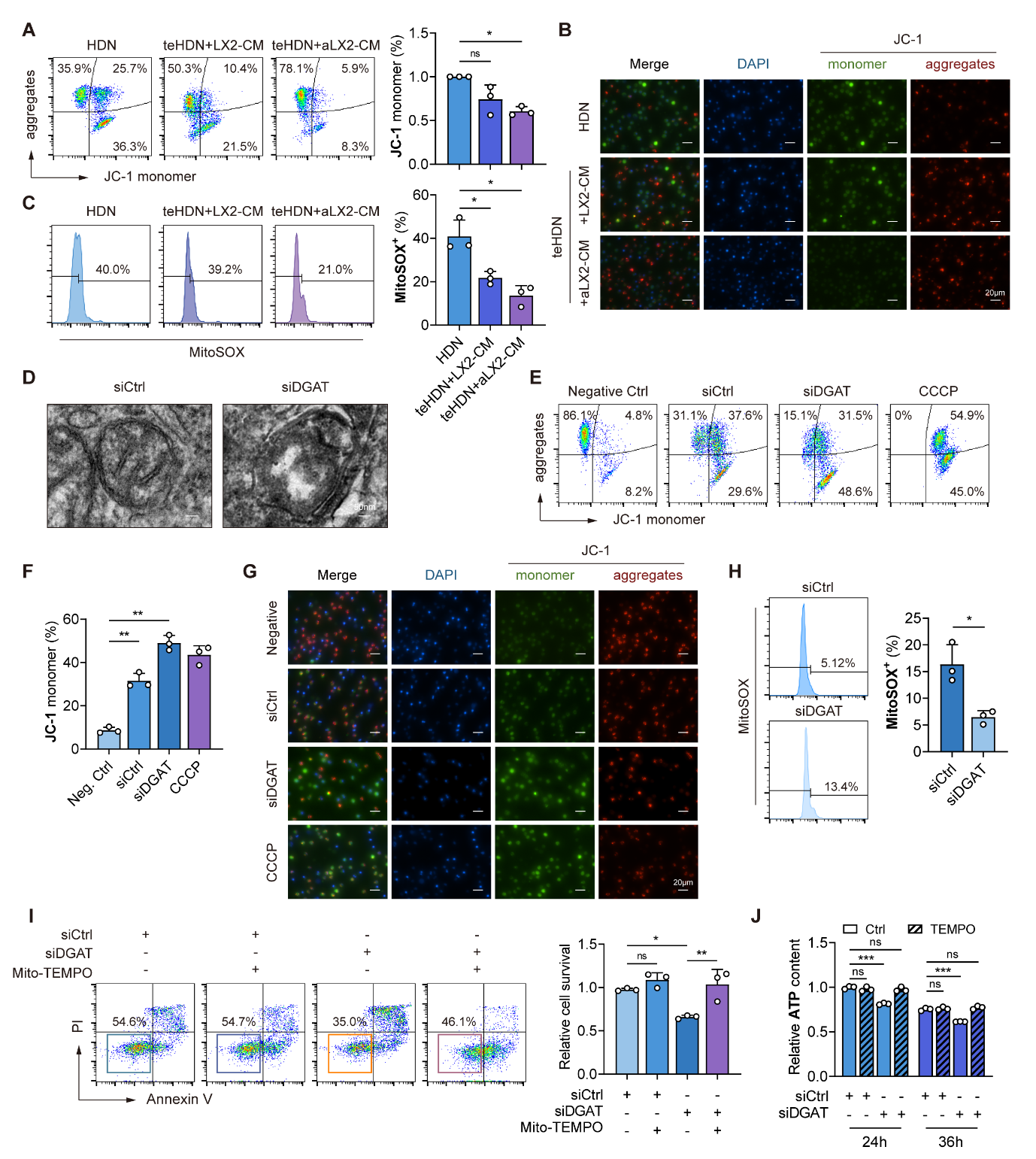


**Fig. S5 DGAT inhibition leads to mitochondrial damage and elevated reactive oxygen species.** (**A-B**) Flow cytometry (*n*=3) and representative fluorescence imaging (representative of *n*=5) of JC-1 monomer and aggregates in HDNs, and teHDNs treated with LX2-CM or aLX2-CM, showing their mitochondrial membrane potential. Scale bar, 20μm. (**C**) Flow cytometry of MitoSOX staining in HDNs, and teHDNs treated with LX2-CM or aLX2-CM, showing their mitochondrial superoxide levels (*n*=3). (**D**) Representative TEM imaging of the mitochondria in teHDNs with/without siRNA knockdown of DGAT1/2. Scale bar, 50nm. (**E-G**) Flow cytometry (*n*=3) and representative fluorescence imaging (representative of *n*=5) of JC-1 monomer and aggregates in teHDNs with/without siRNA knockdown of DGAT1/2. Scale bar, 20μm. (**H**) Flow cytometry of MitoSOX staining in teHDNs with/without siRNA knockdown of DGAT1/2 (*n*=3). (**I-J**) Relative cell survival of teHDNs with/without siRNA knockdown of DGAT1/2 treated with Mito-TEMPO detected by apoptosis assay and relative ATP levels (*n*=3).


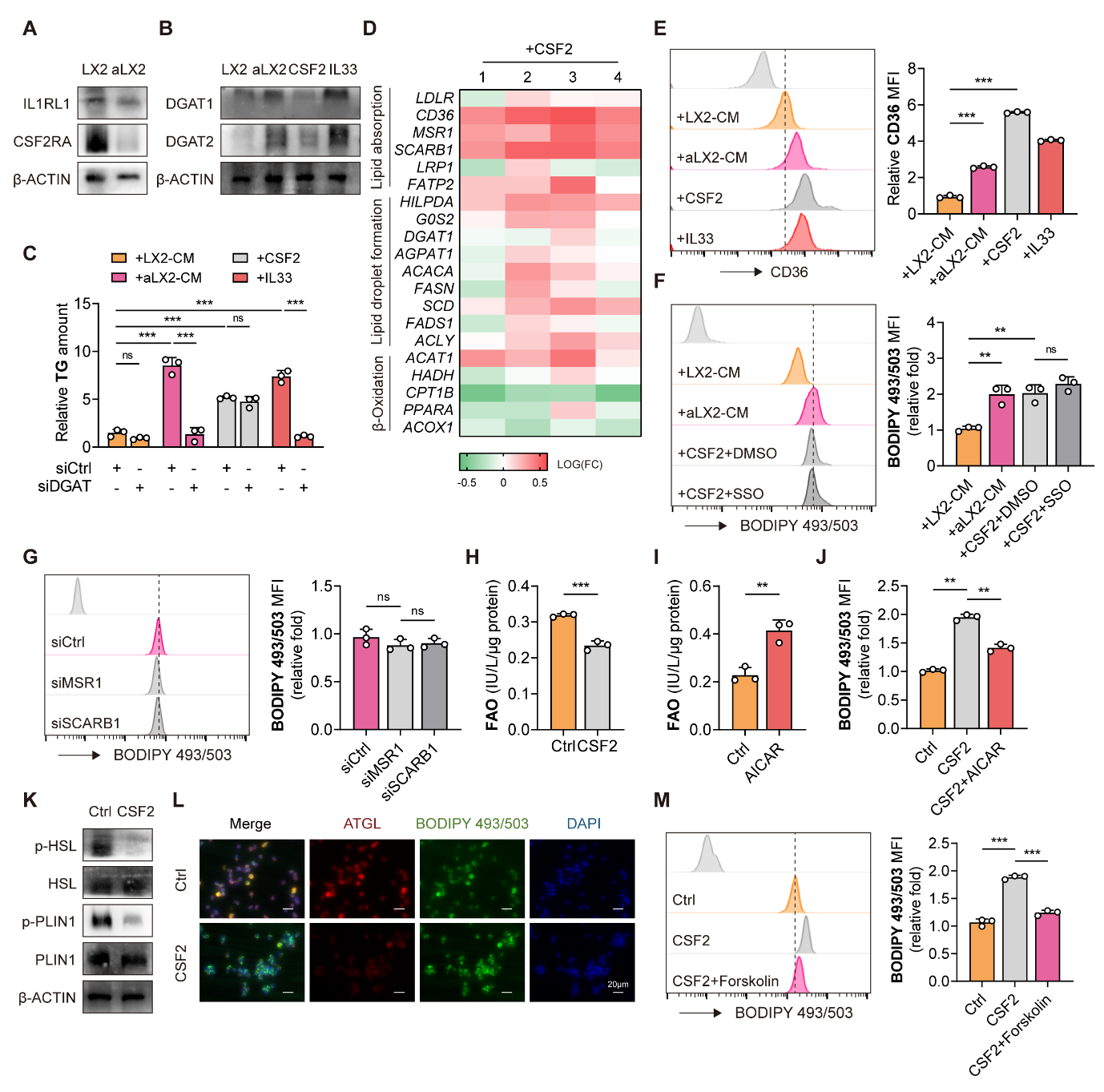


**Fig. S6 CSF2 increases the lipid content of teHDNs by regulating fatty acid oxidation.** (**A**) Immunoblots of IL1RL1 and CSF2RA in teHDNs treated with LX2-CM/aLX2-CM. (**B**) Immunoblots of DGAT1, DGAT2 and PPARG in designated teHDNs treated with CSF2 or IL33. (**C**) Relative TG amount in teHDNs with/without siRNA knockdown of DGAT1/2 treated with CSF2 or IL33. (**D**) Relative mRNA expression of genes related to lipid absorption, lipid droplet formation and β-oxidation in teHDNs treated with CSF2 (*n*=4). (**E**) CD36 expression of designated teHDNs treated with CSF2 or IL33 detected by flow cytometry (*n*=3). (**F**) Lipid levels in CSF2-treated teHDNs after the addition of SSO (*n*=3). (**G**) Lipid levels in teHDNs with/without siRNA knockdown of MSR1/SCARB1 (*n*=3). (**H-I**) The fatty acid oxidation (FAO) level of designated teHDNs treated with CSF2 or AICAR (*n*=3). (**J**) Lipid levels in CSF2-treated teHDNs after the addition of AICAR (*n*=3). (**K**). Immunoblots of p-HSL/HSL and p-PLIN1/PLIN1 in teHDNs treated with CSF2. (**L**) Representative fluorescence imaging (representative of *n*=5) of intracellular localization of ATGL. (**M**) Lipid levels in CSF2-treated teHDNs after the addition of forskolin (*n*=3).


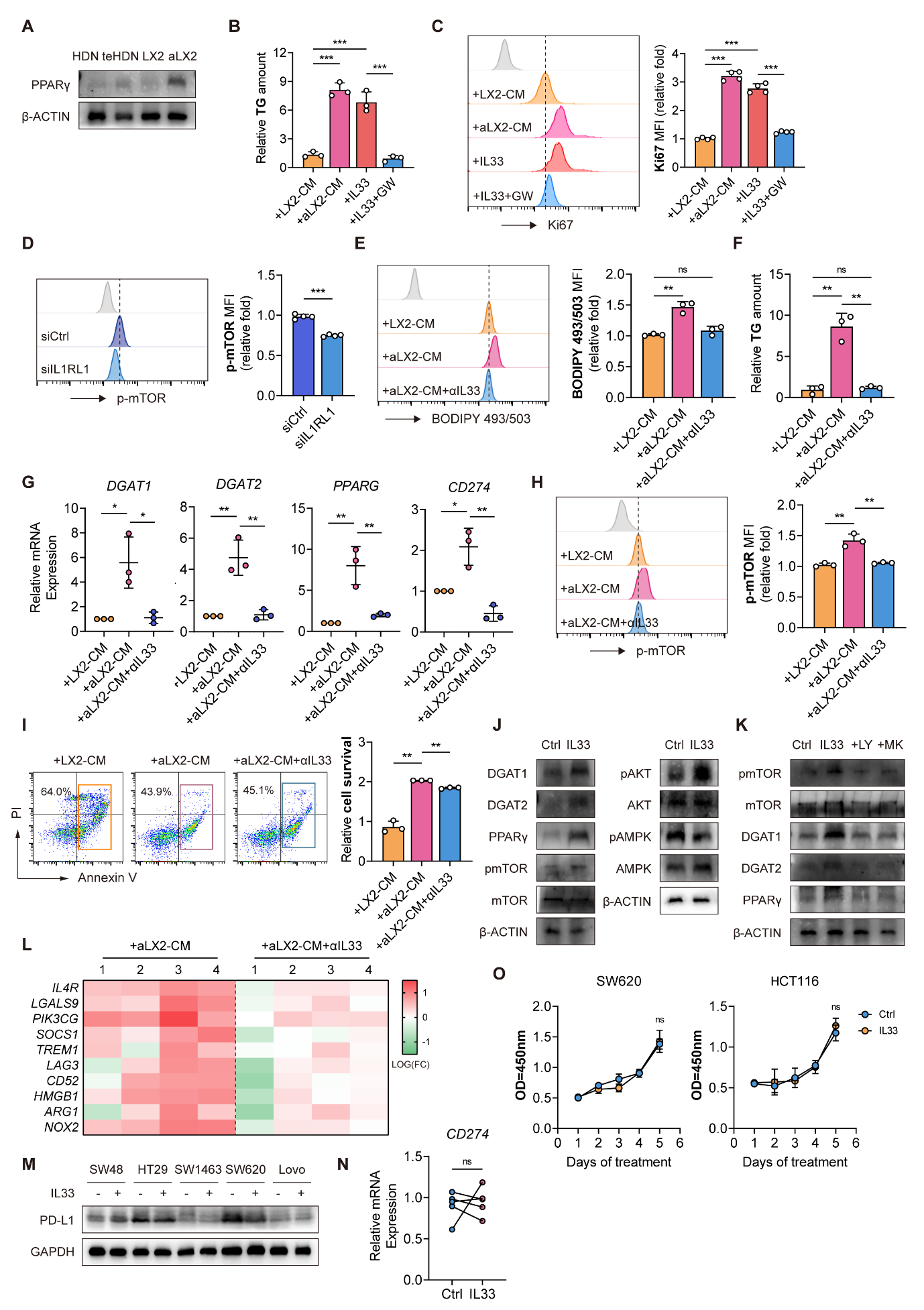


**Fig. S7 IL33 maintains the survival and immunosuppressive function of lipid-laden M-LDNs by regulating mTOR/PPARγ pathway.** (**A**) Immunoblots of PPARγ in teHDNs treated with LX2-CM/aLX2-CM. (**B**) Relative TG amount in teHDNs treated with GW9662 (*n*=3). (**C**) Relative Ki67 expression of teHDNs treated with GW9662 (*n*=3). (**D**) Relative p-mTOR expression of teHDNs with/without siRNA knockdown of IL1RL1 (*n*=3). (**E-F**) Lipid levels and in TG amount teHDNs treated with IL33 neutralizing antibodies (*n*=3). (**G**) Relative mRNA expression of DGAT1, DGAT2, PPARG and CD274 in teHDNs treated with IL33 neutralizing antibodies (*n*=3). (**H**) Relative p-mTOR expression of teHDNs treated with IL33 neutralizing antibodies (*n*=3). (**I**) Relative cell survival of teHDNs treated with IL33 neutralizing antibodies (*n*=3). (**J**) Immunoblots of pAKT, pAMPK and pmTOR in teHDNs after the treatment of IL33. (**K**) Immunoblots of DGAT1, DGAT2, PPARγ and pmTOR in teHDNs after the treatment of IL33 with/without PI3K inhibitor LY294002 (LY) or AKT inhibitor MK-2206 (MK). (**L**) Relative mRNA expression of immune suppression related genes in teHDNs treated with IL33 neutralizing antibodies (*n*=4). (**M-N**) Representative immunoblotting and relative mRNA expression of PD-L1 in IL33-treated colorectal cancer cells. (**O**) Cell growth curves of SW620 and HCT116 treated with IL33 (*n*=3).


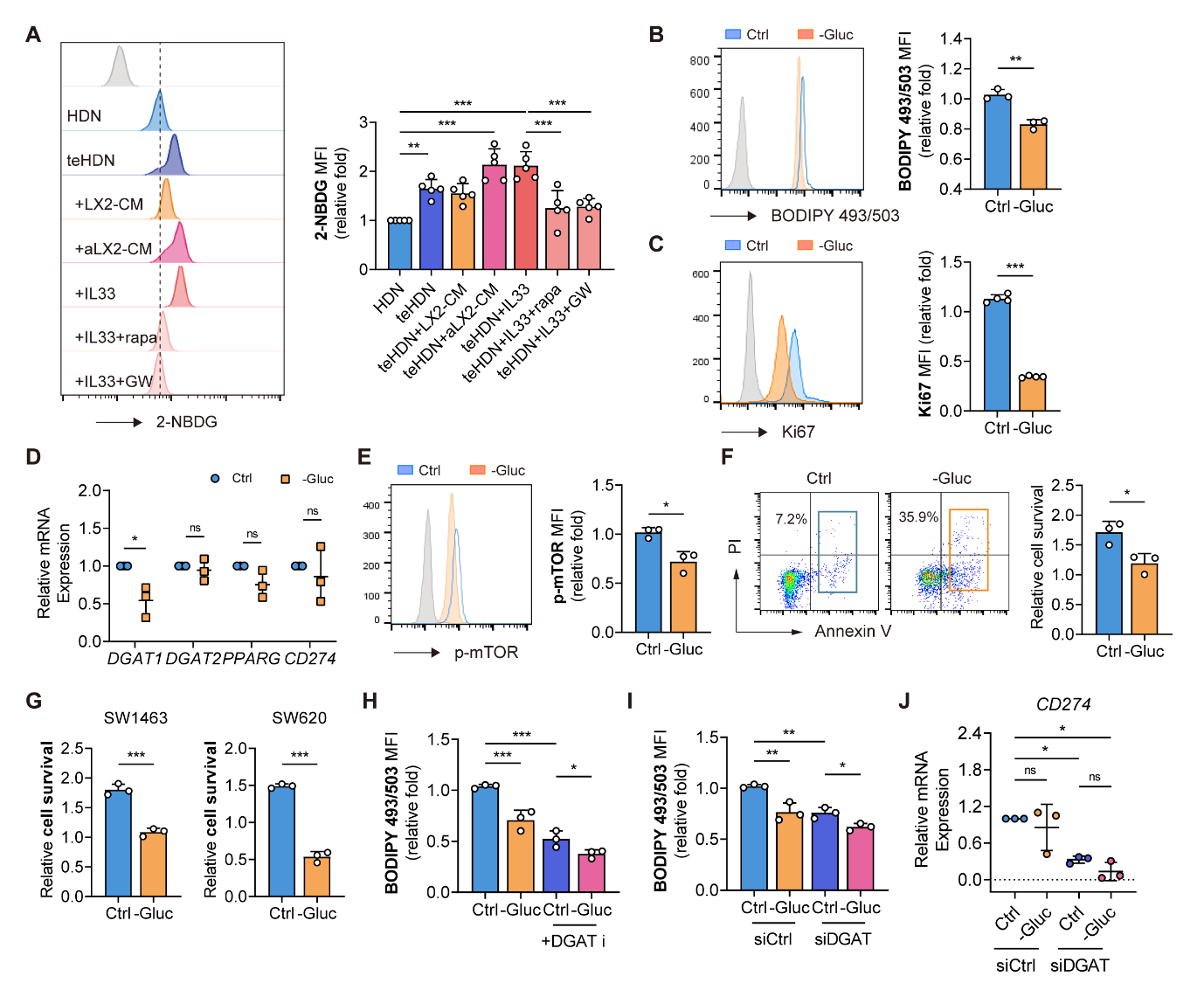


**Fig. S8 Glucose synergizes with IL33 to promote lipid metabolism and immunosuppressive function of M-LDNs.** (**A**) Relative glucose uptake levels (2-NBDG staining) of designated neutrophils (*n*=4). (**B**) Lipid levels in teHDNs cultured in RPMI 1640 without glucose (*n*=3). (**C**) Relative Ki67 expression of teHDNs cultured in RPMI 1640 without glucose (*n*=3). (**D**) Relative mRNA expression of DGAT1, DGAT2, PPARG and CD274 in teHDNs cultured in RPMI 1640 without glucose (*n*=3). (**E**) Relative p-mTOR expression of teHDNs cultured in RPMI 1640 without glucose (*n*=3). (**F**) Relative cell survival of teHDNs cultured in RPMI 1640 without glucose (*n*=3). (**G**) Relative cell survival of SW1463 and SW620 cocultured with teHDNs cultured in RPMI 1640 without glucose (*n*=3). (**H-I**) Lipid levels in teHDNs cultured in RPMI 1640 without glucose treated with DGAT inhibitors or transfected with DGAT1/2 siRNA (*n*=3). (**J**) Relative mRNA expression of CD274 in teHDNs cultured in RPMI 1640 without glucose transfected with DGAT1/2 siRNA (*n*=3).


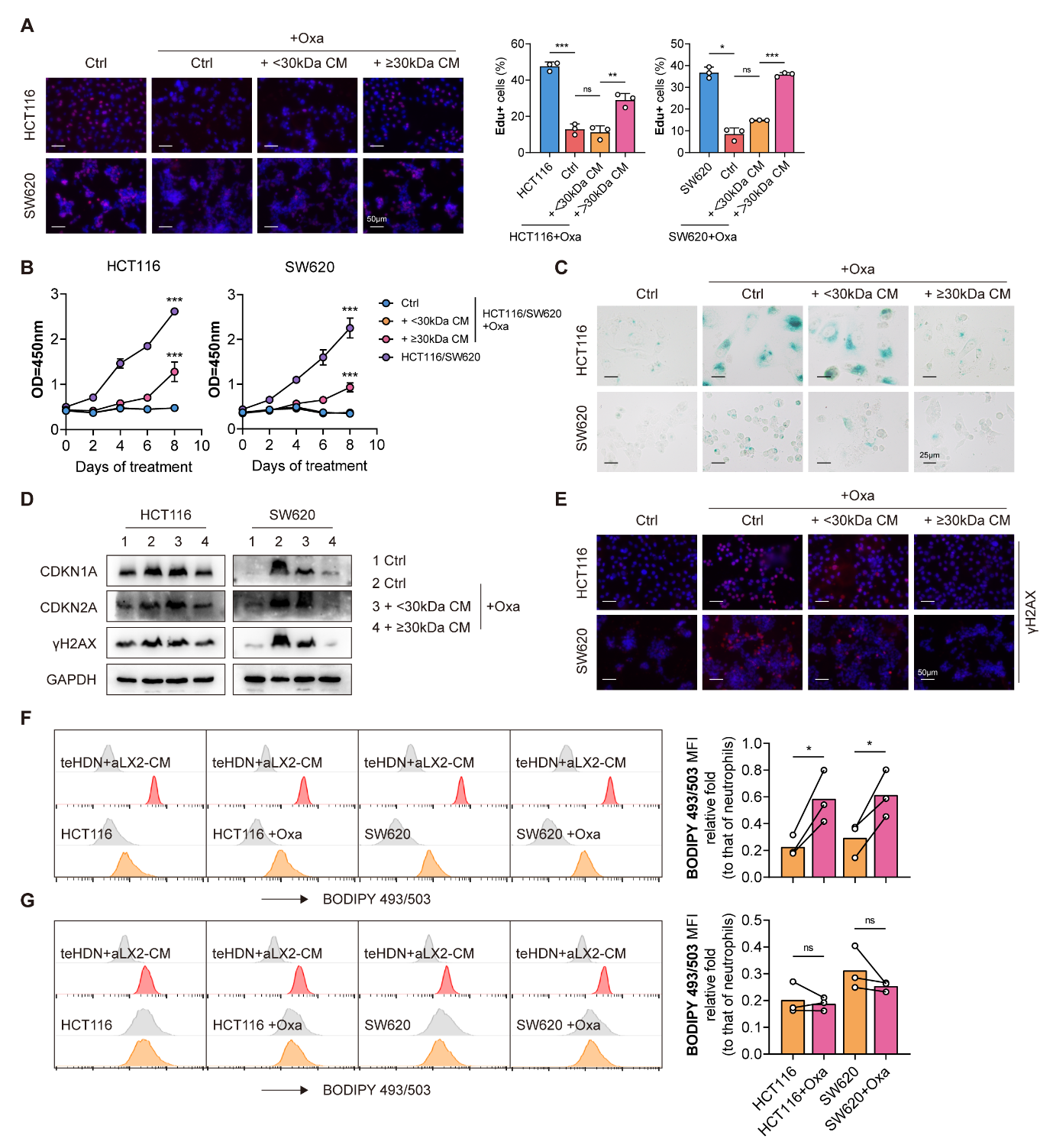


**Fig. S9** **Dormant tumor cells uptake lipids from lipid-laden teHDNs.** (**A**) Representative fluorescence images and relative EdU^+^ cell proportions of neutrophil-CM-treated dormant HCT116 and SW620 (*n*=3). Scale bar, 50μm. (**B**) Cell growth curves of dormant HCT116 and SW620 treated with neutrophil CM (*n*=3). (**C**) Representative images of senescence-associated β-galactosidase staining in neutrophil-CM-treated dormant HCT116 and SW620 (*n*=5 images in total). Scale bar, 25μm. (**D**) Representative immunoblotting of CDKN1A, CDKN2A and γH2AX in neutrophil-CM-treated dormant HCT116 and SW620. (**E**) Representative fluorescence images of γH2AX in neutrophil-CM-treated dormant HCT116 and SW620. (**F**-**G**) Lipid levels in HCT116 and SW620 co-cultured with lipid-fluorescently labeled neutrophils or treated them with neutrophil-CM (*n*=3).


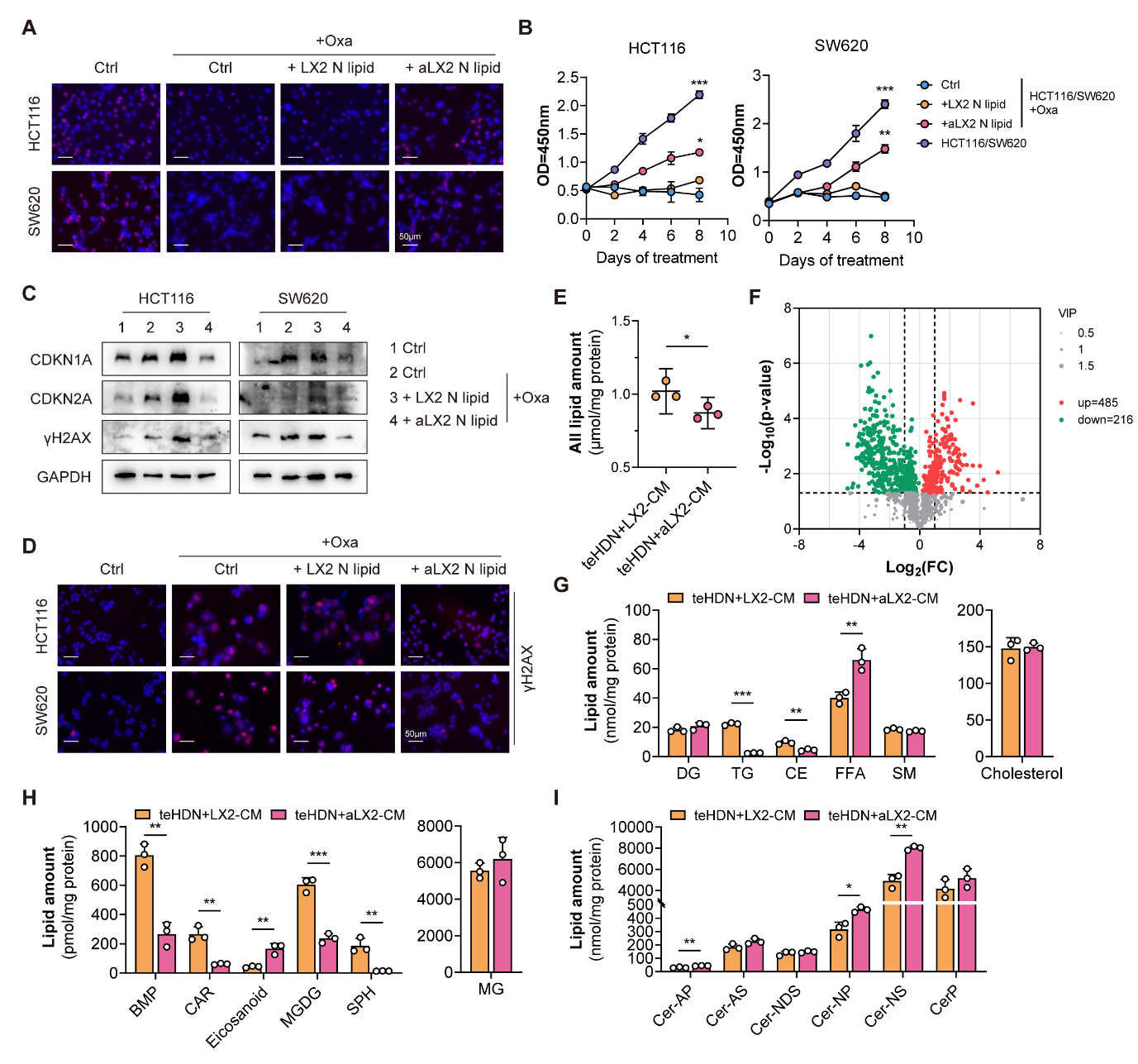


**Fig. S10** **Lipids from lipid-laden neutrophils promote the reactivation of dormant tumor cells.** (**A**) Representative fluorescence images and relative EdU^+^ cell proportions of dormant HCT116 and SW620 treated with extracted neutrophil lipid components (*n*=3). Scale bar, 50μm. (**B**) Cell growth curves of dormant HCT116 and SW620 treated with extracted neutrophil lipid components (*n*=3). (**C**) Representative immunoblotting of CDKN1A, CDKN2A and γH2AX in dormant HCT116 and SW620 treated with extracted neutrophil lipid components. (**D**) Representative fluorescence images of γH2AX in dormant HCT116 and SW620 treated with extracted neutrophil lipid components. (**E**) Total lipid amount of dormant HCT116 treated with designated neutrophils (*n*=3). (**F**) Volcano plots showing fold change and *P* value for the comparison of individual lipid species in dormant HCT116 treated with designated neutrophils (*n*=3). (**G-I**) Levels of representative individual lipid species in dormant HCT116 treated with designated neutrophils (*n*=3). DG, diglyceride. TG, triglyceride. CE, cholesteryl ester. FFA, free fatty acid. SM, sphingomyelin. BMP, Bismonooleoylglycerophosphate. CAR, carnitine. MGDG, Monogalactosyldiacylglycerol. SPH, Sphingosine. MG, monoglyceride. Cer, ceramide.


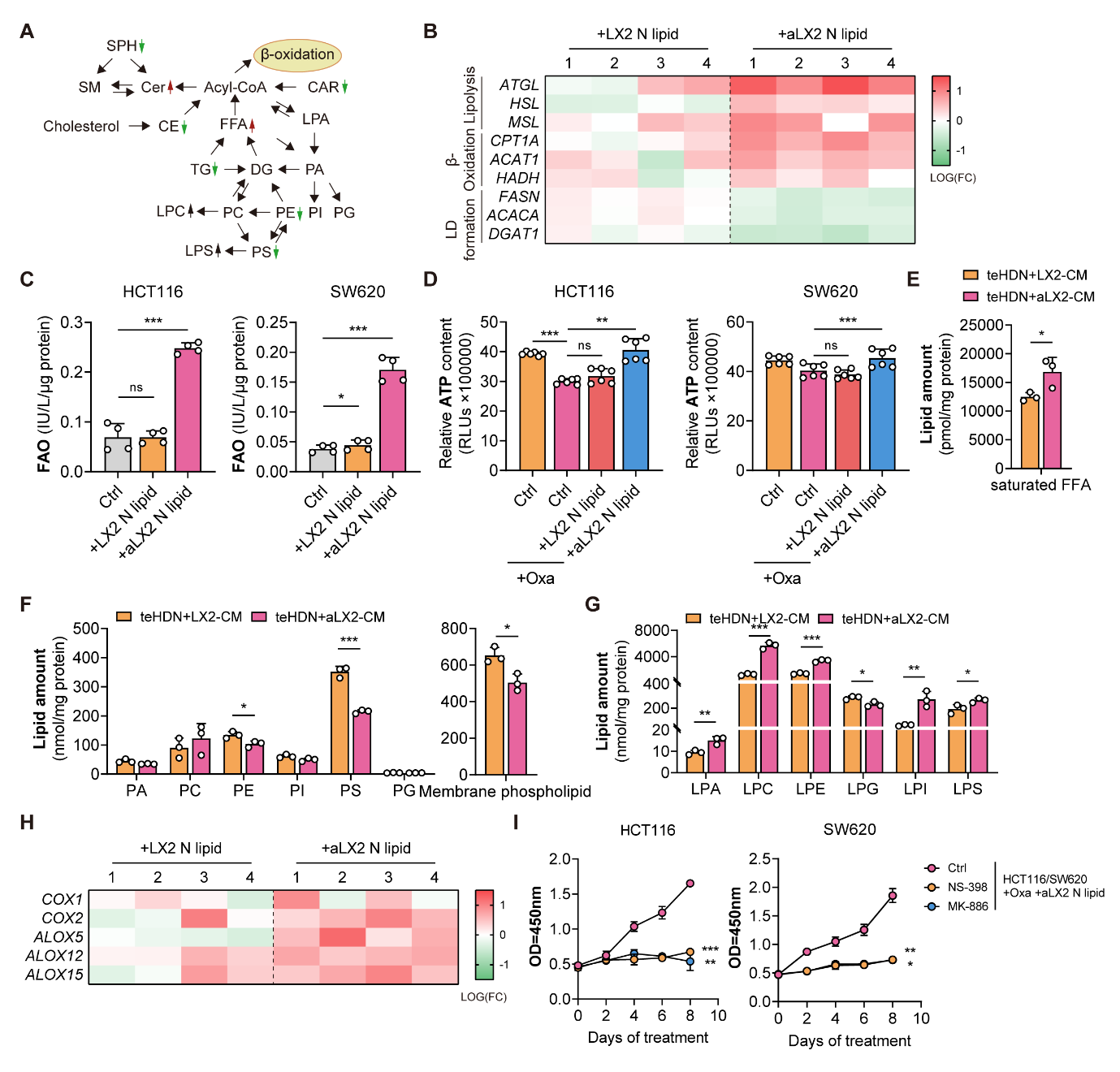


**Fig. S11** **Dormant colorectal cancer cells exit quiescence by enhancing fatty acid oxidation and promoting eicosanoid synthesis.** (**A**) Summary of the transformation of different lipid species (**B** & **H**) Relative mRNA expression of lipid and eicosanoid metabolism related genes in dormant tumor cells treated with extracted neutrophil lipid components (*n*=4). (**C-D**) The FAO and ATP level of dormant HCT116 treated with designated neutrophils (*n*=4). (**E-G**) Levels of representative individual lipid species in dormant HCT116 treated with designated neutrophils (*n*=3). PA, phosphatidic acid. PC, phosphatidylcholine. PE, phosphatidylethanolamine. PI, phosphatidylinositol. PS, phosphatidylserine. PG, phosphatidylglycerol. LPA, lysophosphatidic acid. LPC, lysophosphatidylcholine. LPE, lysophosphatidylethanolamine. LPG, lysophosphatidylglycerol. LPI, lysophosphatidylinositol. LPS, lysophosphatidylserine. (**I**) Cell growth curves of dormant HCT116 and SW620 treated with extracted neutrophil lipid components in addition of COX-2 inhibitor (NS-398) or LOX inhibitor (MK-886) (*n*=3).


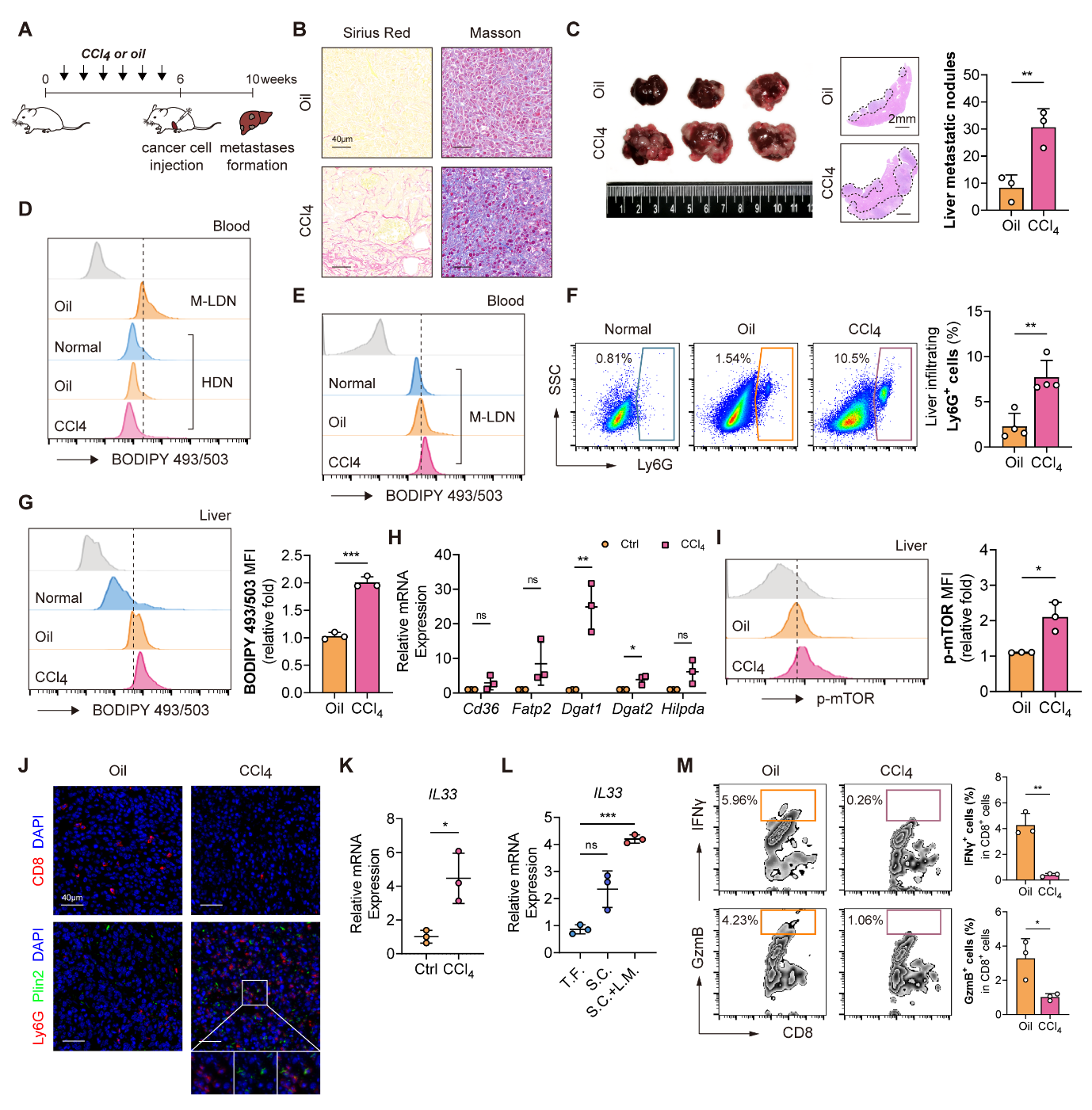


**Fig. S12 Activation of hepatic stellate cells can promote lipid accumulation of liver-infiltrating M-LDNs and CD8^+^ T cell inhibition.** (**A**) Therapeutic scheme for CCl_4_ treatment in BALB/c mice with liver metastasis after intrasplenic transplantation with CT26. Tumors were harvested and photographed at the end of experiments. (**B**) The representative pictures of CCl_4_-treated liver stained with Sirius Red and Masson (representative of *n*=5). Scale bar, 40μm. (**C**) Photos and H&E staining of the livers in mice after CCl_4_ treatments (*n*=3). Scale bar (right), 2mm. (**D-E**) Lipid levels in HDNs and M-LDNs from peripheral blood in CCl_4_-treated mice (*n*=3). (**F**) Proportions of liver infiltrating Ly6G^+^ neutrophils in CCl_4_-treated mice (*n*=3). (**G**) Lipid levels in liver infiltrating M-LDNs after CCl_4_ treatments (*n*=3). (**H**) Relative mRNA expression of lipid metabolism genes in liver infiltrating M-LDNs after CCl_4_ treatments (*n*=3). (**I**) Relative p-mTOR expression of liver infiltrating M-LDNs after CCl_4_ treatments (*n*=3). (**J**) Representative fluorescence images of Ly6G, Plin2 and CD8 in CCl_4_-treated mouse liver metastasis (*n*=5 images in total). Scale bar, 40μm. (**K**) Relative mRNA expression of IL33 in HSCs of CCl_4_-treated mice (*n*=3). (**L**) Relative mRNA expression of IL33 in HSCs of different tumor-bearing mice (*n*=3). (**M**) Proportions of liver infiltrating IFNγ^+^ and GzmB^+^ CD8^+^ T cells in CCl_4_-treated mice (*n*=3).


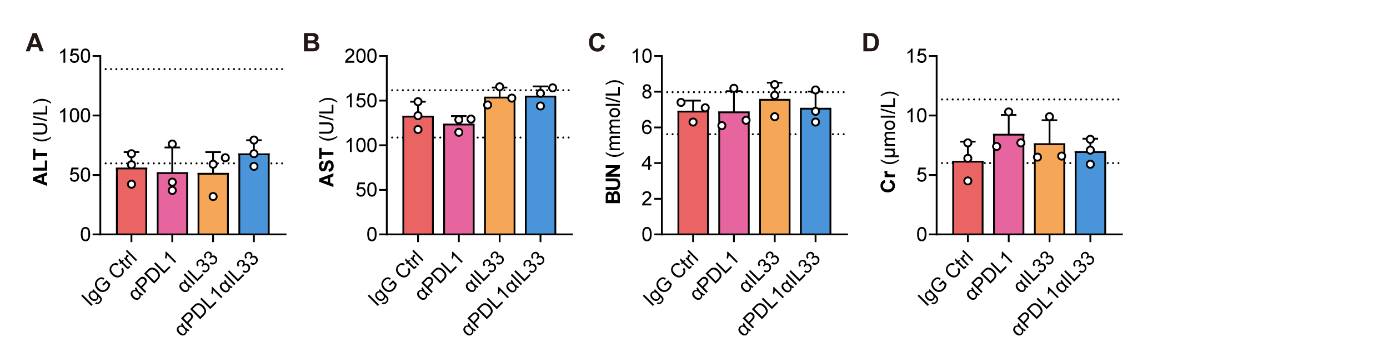


**Fig. S13** **Liver and kidney function parameters in mice following combination therapy.** (**A-D**) The levels of ALT, AST, BUN, Cr in mice treated with anti-IL33 or/and anti-PD-L1 neutralizing antibody.


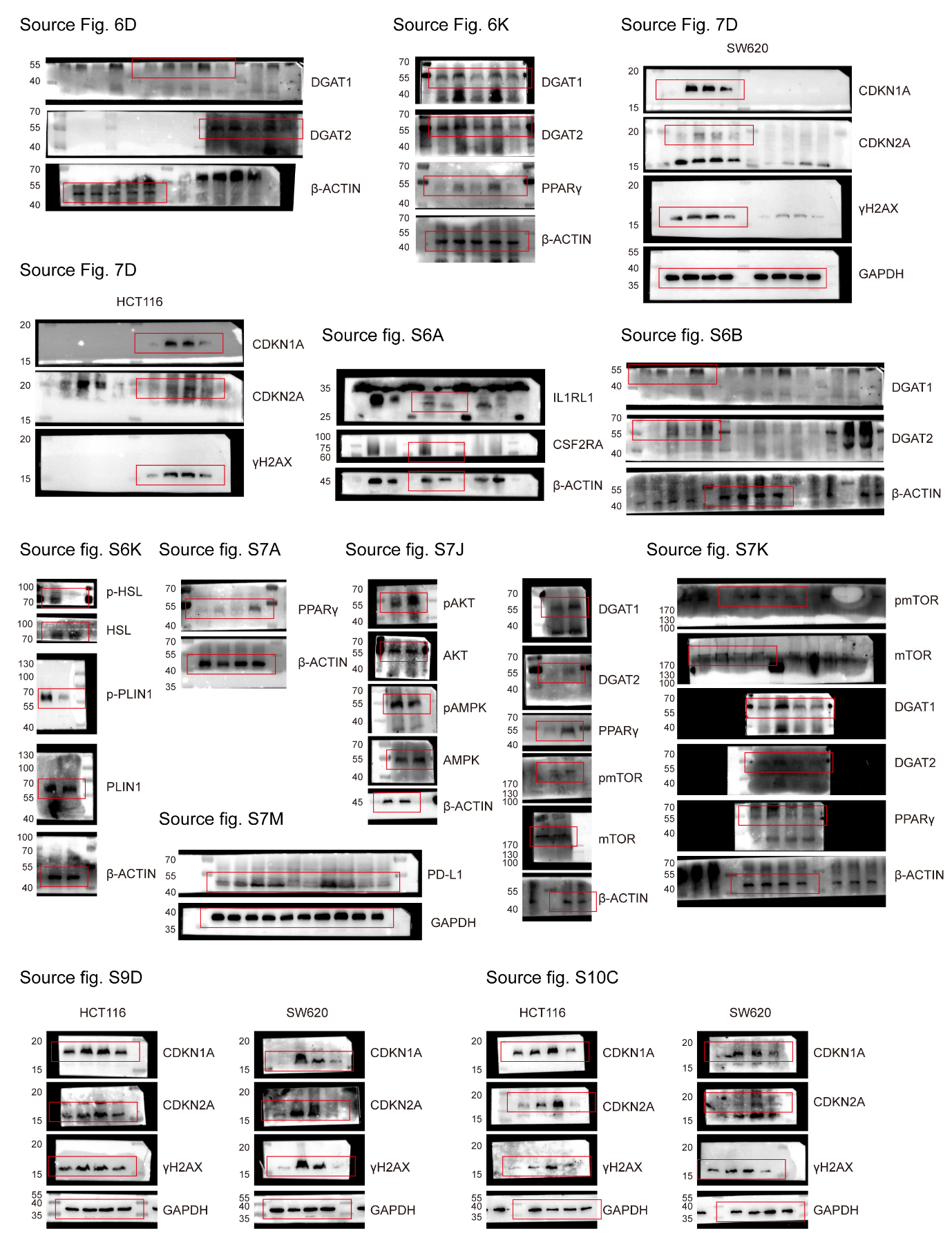


**Fig. S14 Unprocessed original images of western blots.**

**Supplementary Tables**

**Table S1 Summary of clinical information related to Figure 1A and 1B.**

| **Sample #** | **Sex** | **Age [year]** | **Tumor location*** | **T stage** | **N stage** | **M stage** | **Stage** | **MSI status** | **Tumor different-iation**** |
| --- | --- | --- | --- | --- | --- | --- | --- | --- | --- |
| P031901 | F | 69 | R | 4 | 0 | 0 | Ⅰ | MSS | P |
| P031902 | F | 63 | R | 2 | 1 | 0 | Ⅱ | MSS | M |
| P031903 | M | 58 | D | 1 | 0 | 0 | Ⅰ | MSS | M |
| P032504 | M | 50 | A | 2 | 0 | 0 | Ⅰ | MSS | M |
| P032602 | M | 71 | S | 2 | 0 | 0 | Ⅱ | MSS | M |
| P032801 | M | 72 | S | 3 | 1 | 0 | Ⅲ | MSS | M |
| P032802 | F | 67 | S | 1 | 0 | 0 | Ⅰ | MSS | M |
| P0331 | F | 63 | S | 4 | 2 | 1 | Ⅳ | MSS | P |
| P0401 | F | 65 | R | 4 | 1 | 1 | Ⅳ | MSS | / |
| P040101 | M | 83 | S | 3 | 0 | 0 | Ⅱ | MSS | M |
| P0403 | F | 53 | S | 3 | 1 | 0 | Ⅲ | MSS | M |
| P0407 | M | 68 | D | 2 | 0 | 0 | Ⅱ | MSI-H | M |
| P040802 | M | 47 | A | 3 | 0 | 0 | Ⅱ | MSS | / |
| P0410 | M | 55 | S | 3 | 1 | 0 | Ⅲ | MSS | M-P |
| P0417 | M | 57 | D | 3 | 0 | 0 | Ⅱ | MSS | M |
| P041902 | F | 53 | R | 4 | 0 | 0 | Ⅱ | MSS | M |
| P042501 | M | 64 | S | 3 | 0 | 0 | Ⅱ | MSS | M |
| P0506 | F | 63 | A | 4 | 2 | 1 | Ⅳ | MSS | M |
| P0509 | M | 67 | R | 2 | 0 | 0 | Ⅰ | MSS | M |
| P0512 | M | 81 | R | 4 | 1 | 1 | Ⅳ | MSS | / |
| P052301 | M | 82 | R | 3 | 0 | 0 | Ⅱ | MSS | M |
| P052302 | M | 55 | R | 3 | 2 | 0 | Ⅲ | MSS | M |
| P0528 | F | 73 | S | 3 | 0 | 0 | Ⅱ | MSS | M |
| P060401 | M | 80 | T | 3 | 0 | 0 | Ⅱ | MSS | M-P |
| P060402 | F | 55 | R | 1 | 0 | 0 | Ⅰ | MSS | M |
| P060601 | F | 56 | S | 3 | 1 | 0 | Ⅲ | MSS | M |
| P060602 | M | 41 | S | 2 | 0 | 0 | Ⅰ | MSS | M |
| P0619 | M | 78 | R | 3 | 1 | 1 | Ⅳ | MSS | M-P |
| P062102 | M | 72 | R | 3 | 1 | 0 | Ⅲ | MSS | M |
| P0626 | M | 90 | A | 4 | 2 | 1 | Ⅳ | MSS | P |
| P0828 | M | 62 | D | 3 | 1 | 0 | Ⅲ | MSS | M |
| P0903 | F | 84 | A | 3 | 1 | 0 | Ⅲ | MSS | M |
| P0905 | M | 71 | D | 4 | 1 | 0 | Ⅲ | MSS | M |
| P0913 | M | 72 | R | 4 | 1 | 0 | Ⅲ | MSS | M |
| P0919 | M | 64 | D | 4 | 2 | 1 | Ⅳ | MSS | P |

* A, Ascending colon; T, Transverse colon; D, Descending colon; S, Sigmoid colon; R, Rectum.

** M, moderately; P, poorly.

**Table S2 Summary of clinical information related to Figure 3 and S3.**

| **Sample #** | **Sex** | **Age [year]** | **Tumor location** | **T stage** | **N stage** | **M stage** | **Stage** | **MSI status** | **Tumor different-iation*** |
| --- | --- | --- | --- | --- | --- | --- | --- | --- | --- |
| P112202 | M | 74 | S | 1 | 0 | 0 | Ⅰ | MSS | M |
| P1123 | M | 85 | R | 3 | 2 | 0 | Ⅲ | MSS | M |
| P1226 | M | 29 | S | 4 | 1 | 1 | Ⅳ | MSS | M |
| P1229 | M | 50 | D | 3 | 0 | 0 | Ⅱ | MSS | M |

* D, Descending colon; S, Sigmoid colon; R, Rectum.

** M, moderately.

**Table S3 Primer sequences for qPCR.**

| **Gene** |  | **Sequence** |
| --- | --- | --- |
| hAcaa2 | F | AGACAATGCAGGTAGACGAGC |
|  | R | ACCCATGATAGAGGGATCACATC |
| hAcaca | F | ATGTCTGGCTTGCACCTAGTA |
|  | R | CCCCAAAGCGAGTAACAAATTCT |
| hAcat1 | F | AAGGCAGGCAGTATTGGGTG |
|  | R | ACATCAGTTAGCCCGTCTTTTAC |
| hAcly | F | ATCGGTTCAAGTATGCTCGGG |
|  | R | GAAGGGCTCGATCAGAAAGTTC |
| hAcox1 | F | GGAACTCACCTTCGAGGCTTG |
|  | R | TTCCCCTTAGTGATGAGCTGG |
| hActa2 | F | CTTCGTTACTACTGCTGAGCGTGAG |
|  | R | CCCATCAGGCAACTCGTAACTCTTC |
| hActb | F | CATGTACGTTGCTATCCAGGC |
|  | R | CTCCTTAATGTCACGCACGAT |
| hAgpat1 | F | GAGGGAACGAGAAACCACAAT |
|  | R | AGTCTTGGTAGGAGGACATGAC |
| hAlox5 | F | CTCAAGCAACACCGACGTAAA |
|  | R | CCTTGTGGCATTTGGCATCG |
| hAlox12 | F | ACCAGTTCCTCAATGGTGCC |
|  | R | TCCTCGGATCACGTTGGCT |
| hAlox15 | F | GGGCAAGGAGACAGAACTCAA |
|  | R | CAGCGGTAACAAGGGAACCT |
| hArg1 | F | ACACTCCACTGACAACCACA |
|  | R | TCCACGTCTCTCAAGCCAAT |
| hC3 | F | GGGGAGTCCCATGTACTCTATC |
|  | R | GGAAGTCGTGGACAGTAACAG |
|  |  |  |
| hC5 | F | CAGGGAAGGTTACCGAGCAAT |
|  | R | TCAGATGTTCTCCCACTAGCA |
| hCcl2 | F | CAGCCAGATGCAATCAATGCC |
|  | R | TGGAATCCTGAACCCACTTCT |
| hCcl5 | F | CCAGCAGTCGTCTTTGTCAC |
|  | R | CTCTGGGTTGGCACACACTT |
| hCcl7 | F | TGCTCAGCCAGTTGGGATTA |
|  | R | GCTACTGGTGGTCCTTCTGT |
| hCd36 | F | CTTTGGCTTAATGAGACTGGGAC |
|  | R | GCAACAAACATCACCACACCA |
| hCd52 | F | ACAGCCACGAAGATCCTACC |
|  | R | CGTGTCACCTCAACTGAAGC |
| hCol1a1 | F | GTGCGATGACGTGATCTGTGA |
|  | R | CGGTGGTTTCTTGGTCGGT |
| hCox2(Ptgs2) | F | ATGCTGACTATGGCTACAAAAGC |
|  | R | TCGGGCAATCATCAGGCAC |
| hCpt1b | F | CATGTATCGCCGTAAACTGGAC |
|  | R | TGGTAGGAGCACATAGGCACT |
| hCpt2 | F | CATACAAGCTACATTTCGGGACC |
|  | R | AGCCCGGAGTGTCTTCAGAA |
| hCsf2 | F | TCCTGAACCTGAGTAGAGACAC |
|  | R | TGCTGCTTGTAGTGGCTGG |
| hCsf2ra | F | AATGAACTGTACCTGGGCGA |
|  | R | CCAATTTCTCGGCTGGTTCC |
| hCxcl1 | F | AGGCAGGGGAATGTATGTGC |
|  | R | AGCCCCTTTGTTCTAAGCCAG |
| hCxcl2 | F | CCACCTGGATTGCGCCTAA |
|  | R | TGCTCTAACACAGAGGGAAACAC |
| hCxcl5 | F | AGCTGCGTTGCGTTTGTTTAC |
|  | R | TGGCGAACACTTGCAGATTAC |
| hCxcl8 | F | ACTGAGAGTGATTGAGAGTGGAC |
|  | R | AACCCTCTGCACCCAGTTTTC |
| hCxcl10 | F | GTGGCATTCAAGGAGTACCTC |
|  | R | TGATGGCCTTCGATTCTGGATT |
| hCybb(Nox2) | F | AAAGAGGGTTGGAGGTGGAG |
|  | R | ACGGAAAATAAGGGGTGGGT |
| hCyp51a1 | F | GCAATCCAGAAACGCAGACA |
|  | R | TTGAGGATGTATGCTGCCCT |
| hDes | F | GAGACCATCGCGGCTAAGAAC |
|  | R | GTGTAGGACTGGATCTGGTGT |
| hDgat1 | F | CAATCTGACCTACCGCGATCT |
|  | R | TCGATGATGCGTGAGTAGTCC |
| hDgat2 | F | GTGAGGGCAGTAGTAGGCAT |
|  | R | ATGTCATCAGCCACCCAAGA |
| hEgf | F | TGTCCACGCAATGTGTCTGAA |
|  | R | CATTATCGGGTGAGGAACAACC |
| hFads1 | F | CTACCCCGCGCTACTTCAC |
|  | R | CGGTCGATCACTAGCCACC |
| hFasn | F | ACAGCGGGGAATGGGTACT |
|  | R | GACTGGTACAACGAGCGGAT |
| hFatp2 | F | CGGATGTATTGTGGCTGGTG |
|  | R | ATTTCCCAGTGCCAGTCTCA |
| hG0s2 | F | CTCTCCAGAAGCAAGCCCT |
|  | R | CCCCTCTGTCTCTCCATTCC |
| hGapdh | F | CCCTCAACGACCACTTTGTC |
|  | R | AGGGGAGATTCAGTGTGGTG |
| hSlc2a1(Glut1) | F | TGCCTTCTTCACCCAGCTAA |
|  | R | AAGAGATGGGAAGGGGCAAA |
| hSlc2a3(Glut3) | F | CACGTCTCTCTGGTCCTTGT |
|  | R | AATAACCAAGCGACCCAGGA |
| hHadh | F | ACCCTGAGCACCATAGCGA |
|  | R | CAGCGAATCGGTCTTGTCTGG |
| hHilpda | F | TCAGAAGGAGAAGGCAGCTC |
|  | R | GCTCTGTGTTGGCTAGTTGG |
| hHk1 | F | AAGACTCTAAGGCGCTTGGT |
|  | R | CCTCTTCTTCACCTCCAGCA |
| hHmgb1 | F | TGCTCAGAGAGGTGGAAGAC |
|  | R | CAGAAGAGGAAGAAGGCCGA |
| hHmgcs1 | F | AAGTACCAAGACTCCCTGCC |
|  | R | ATCCAGGCTCCATGTCAGTC |
| hIfng | F | TCGGTAACTGACTTGAATGTCCA |
|  | R | TCGCTTCCCTGTTTTAGCTGC |
| hIgf2 | F | GTGGCATCGTTGAGGAGTG |
|  | R | CACGTCCCTCTCGGACTTG |
| hIl1a | F | AGATGCCTGAGATACCCAAAACC |
|  | R | CCAAGCACACCCAGTAGTCT |
| hIl1b | F | AGCTACGAATCTCCGACCAC |
|  | R | CGTTATCCCATGTGTCGAAGAA |
| hIl17a | F | AGATTACTACAACCGATCCACCT |
|  | R | GGGGACAGAGTTCATGTGGTA |
| hIl1rl1 | F | AACTGGACAGCACCTCTTGA |
|  | R | CTTGCTCATCCTTGACCGTG |
| hIl4 | F | CCAACTGCTTCCCCCTCTG |
|  | R | TCTGTTACGGTCAACTCGGTG |
| hIl4r | F | TCCCTGTTGTAACTGCCCAA |
|  | R | AATGACCACCCTCCCTGAAG |
| hIl6 | F | CCTGAACCTTCCAAAGATGGC |
|  | R | TTCACCAGGCAAGTCTCCTCA |
| hIl33 | F | GTGACGGTGTTGATGGTAAGAT |
|  | R | AGCTCCACAGAGTGTTCCTTG |
| hLag3 | F | CTTTGGAGAAGACAGTGGCG |
|  | R | TATTTGGACTGGGCTGCTGA |
| hLdha | F | ATGGCAACTCTAAAGGATCAGC |
|  | R | CCAACCCCAACAACTGTAATCT |
| hLdlr | F | ACCAACGAATGCTTGGACAAC |
|  | R | ACAGGCACTCGTAGCCGAT |
| hLgals9 | F | TGTTCTCTACTCCCGCCATC |
|  | R | AGGAGTTGTCGATCTGGGTG |
| hLipe(Hsl) | F | TCAGTGTCTAGGTCAGACTGG |
|  | R | AGGCTTCTGTTGGGTATTGGA |
| hLpin1 | F | CCAGCCCAATGGAAACCTCC |
|  | R | AGGTGCATAGGGATAACTTCCTG |
| hLrp1 | F | AACGAGCATAACTGCCTGGG |
|  | R | CGTACACTGAGCACTCATCAAA |
| hMgll(Mgl) | F | TCGTCAGGGATGTGTTGCAG |
|  | R | AGGCGAAATGAGTACCATGCC |
| hMogat2 | F | TCTCTTCGATCTTCCCCGGTA |
|  | R | GATGCCCAGCAAGTTTCCG |
| hMsr1 | F | GCAGTGGGATCACTTTCACAA |
|  | R | AGCTGTCATTGAGCGAGCATC |
| hMvk | F | GTGTGCCTTCTCTCTCCCTT |
|  | R | CCGCTAGGTAGAAGGGAAGG |
| hPdl1(Cd274) | F | TGGCATTTGCTGAACGCATTT |
|  | R | TGCAGCCAGGTCTAATTGTTTT |
| hPfkfb3 | F | ATCTGGACAGGGAGGGAGAT |
|  | R | GGAGGAATCAGTAGACGCCA |
| hPik3cg | F | AGGCAGCAGTGGAGAGATTT |
|  | R | AGGAAGTCAGGGGTTAGCAC |
| hPkm | F | TTTGCTAGTGAGGCCAAGGA |
|  | R | CAGATGATGCCAGTGTTCCG |
| hPnpla2(Atgl) | F | GGCTTCCTCGGCGTCTACTA |
|  | R | TTTACCAGGTTGAAGGAGGGG |
| hPpara | F | TTCGCAATCCATCGGCGAG |
|  | R | CCACAGGATAAGTCACCGAGG |
| hPparg | F | GACCACTCCCACTCCTTTGA |
|  | R | ATGAGGGAGTTGGAAGGCTC |
| hPtgs1(Cox1) | F | CTCTGTGCCTAAAGATTGCCC |
|  | R | GTCTCCATAAATGTGGCCGAG |
| hPtgs2(Cox2) | F | ATGCTGACTATGGCTACAAAAGC |
|  | R | TCGGGCAATCATCAGGCAC |
| hRgs5 | F | GACATGGCCCAGAAAAGAATCC |
|  | R | CACAAAGCGAGGCAGAGAATC |
| hScarb1 | F | CCTATCCCCTTCTATCTCTCCG |
|  | R | GGATGTTGGGCATGACGATGT |
| hScd | F | TCTAGCTCCTATACCACCACCA |
|  | R | TCGTCTCCAACTTATCTCCTCC |
| hSerping1 | F | CCAAGATGCTATTCGTTGAACCC |
|  | R | TGGTGGCTGAATTGGTTGTTG |
| hSlc2a1(Glut1) | F | TGCCTTCTTCACCCAGCTAA |
|  | R | AAGAGATGGGAAGGGGCAAA |
| hSlc2a3(Glut3) | F | CACGTCTCTCTGGTCCTTGT |
|  | R | AATAACCAAGCGACCCAGGA |
| hSocs1 | F | GCTCCTTCCCCTTCCAGATT |
|  | R | TCAAGAGGTGAGAAGGGGTC |
| hSqle | F | TGAGTCCGAGGCCATCTTTT |
|  | R | GAGGGAGGGTCACAGAAACA |
| hSrebf1 | F | CTCAGATACCACCAGCGTCT |
|  | R | TTGCGATGCCTCCAGAAGTA |
| hTgfb1 | F | CAATTCCTGGCGATACCTCAG |
|  | R | GCACAACTCCGGTGACATCAA |
| hTnfa | F | CCTCTCTCTAATCAGCCCTCTG |
|  | R | GAGGACCTGGGAGTAGATGAG |
| hTrem1 | F | ATCATCAGGGTTCCGGTGTT |
|  | R | ACTCCCTGCCTTTTACCTCC |
| hVim | F | AGTCCACTGAGTACCGGAGAC |
|  | R | CATTTCACGCATCTGGCGTTC |
| mCd36 | F | TGATACTATGCCCGCCTCTC |
|  | R | ACATCGTTTCCCACACTCCT |
| mCox2 | F | AGGTCATTGGTGGAGAGGTG |
|  | R | CCTGCTTGAGTATGTCGCAC |
| mCsf2 | F | TTGGAAGCATGTAGAGGCCA |
|  | R | CGCCCTTGAGTTTGGTGAAA |
| mCxcl2 | F | ACTCTCAAGGGCGGTCAAAA |
|  | R | CATCAGGTACGATCCAGGCT |
| mCxcl5 | F | TAGAGCCCCAATCTCCACAC |
|  | R | GGCTATGACTGAGGAAGGGG |
| mDgat1 | F | GATTGTGGGCCGATTCTTCC |
|  | R | GACTAGGAGGAGTGTGCAGG |
| mDgat2 | F | GAGCTGATCTGGTTCCCACT |
|  | R | TGGTGATGGGCTTGGAGTAG |
| mEgf | F | CACTGGACAGCTACACATGC |
|  | R | GGGTTTGATAGCTGCTTCCG |
| mFatp2 | F | AGCTGTGGAGGAGGTTCTTC |
|  | R | GATGATTGATGGTTGCCGCT |
| mGapdh | F | CCCACTCTTCCACCTTCGAT |
|  | R | CTTGCTCAGTGTCCTTGCTG |
| mHilpda | F | GCAGCCGTCTCTCAACTTTT |
|  | R | GTAAGCCTCCCAGAGACTCC |
| mIfng | F | CGGCCTAGCTCTGAGACAAT |
|  | R | TCCTTTTGCCAGTTCCTCCA |
| mIgf2 | F | GGGACGTGTCTACCTCTCAG |
|  | R | ATGACGTTTGGCCTCTCTGA |
| mIl1b | F | GGCTCATCTGGGATCCTCTC |
|  | R | TCATCTTTTGGGGTCCGTCA |
| mIl4 | F | TCTCGAATGTACCAGGAGCC |
|  | R | ACCTTGGAAGCCCTACAGAC |
| mIl17a | F | CAAACACTGAGGCCAAGGAC |
|  | R | CTTTCCCTCCGCATTGACAC |
| mIl33 | F | AGTTCCCACCCGTGATTGAA |
|  | R | GCTCCTTGGAAATCAGCGAG |
| mTgfb1 | F | TCAGACATTCGGGAAGCAGT |
|  | R | TCGAAAGCCCTGTATTCCGT |

**Table S4 Key Resources.**

| **REAGENT or RESOURCE** | **SOURCE** | **IDENTIFIER** |
| --- | --- | --- |
| **Antibodies** | | |
| AMPKa1/AMPKa2 Rabbit pAb | Abclonal | Cat# A12718 |
| Anti-APC MicroBeads | Miltenyi Biotec | Cat# 130-090-855 |
| Anti-Biotin MicroBeads | Miltenyi Biotec | Cat# 130-090-485 |
| Anti-CD8 alpha antibody | Abcam | Cat# ab316778 |
| Anti-GFAP antibody | Abcam | Cat# ab279289 |
| Anti-Ly6G antibody | Abcam | Cat# ab238132 |
| Anti-mouse PD-L1 (B7-H1) antibody | BioXCell | Cat# CP001 |
| Anti-PNPLA2 (ATGL) Recombinant Rabbit mAb | GenuIN Biotech | Cat# 2484 |
| APC anti-human CD3 antibody | Miltenyi Biotec | Cat# 130-113-125 |
| APC anti-human CD36 antibody | BioLegend | Cat# 336207 |
| APC anti-mouse IFN-γ antibody | BD Biosciences | Cat# 562018 |
| APC anti-mouse Ly-6G antibody | BD Biosciences | Cat# 560599 |
| Biotin anti-human CD66b antibody | BioLegend | Cat# 305120 |
| Brilliant Violet 421™ anti-human/mouse Granzyme B antibody | BioLegend | Cat# 396413 |
| CD66b/CEACAM8 Rabbit pAb | Abclonal | Cat# A8113 |
| CDKN1A/p21 Rabbit PolymAb | Abclonal | Cat# A22460 |
| CDKN2A/p16INK4a Rabbit mAb | Abclonal | Cat# A11651 |
| DGAT1 Rabbit pAb | Abclonal | Cat# A6857 |
| DGAT2 Rabbit pAb | Abclonal | Cat# A13891 |
| FITC anti-mouse CD8a antibody | BD Biosciences | Cat# 553030 |
| GAPDH Monoclonal antibody | Proteintech | Cat# 60004-1-Ig |
| GM-CSFRα/CSF2RA Rabbit mAb | Abclonal | Cat# A23001 |
| Goat Anti-Rabbit IgG H&L (Alexa Fluor® 555) | Abcam | Cat# ab150078 |
| Human anti-IL-33 antibody | R&D Systems | Cat# AF3625-SP |
| HSL (D6W5S) XP® Rabbit mAb | Cell Signaling Technology | Cat# 18381 |
| IL1RL1 Rabbit pAb | Abclonal | Cat# A1913 |
| InVivoMAb mouse IgG1 isotype control | BioXCell | Cat# BE0083 |
| Mouse anti-IL-33 antibody | R&D Systems | Cat# AF3626-SP |
| mTOR Rabbit pAb | Abclonal | Cat# A11354 |
| Normal Goat IgG Control | R&D Systems | Cat# AB-108-C |
| Pan-Akt Rabbit mAb | Abclonal | Cat# A22412 |
| PD-L1/CD274 Rabbit mAb | Abclonal | Cat# A19135 |
| PE anti-human CD274 antibody | BD Biosciences | Cat# 561787 |
| PE anti-human CD66b antibody | BioLegend | Cat# 305106 |
| PE anti-mouse/human Ki-67 antibody | BioLegend | Cat# 151209 |
| PE phospho-mTOR (Ser2448) monoclonal antibody | Invitrogen | Cat# 12-9718-41 |
| Perilipin A Rabbit pAb | Abclonal | Cat# A16295 |
| Perilipin 2 (Plin2) Polyclonal antibody | Proteintech | Cat# 15294-1-AP |
| Phospho-Akt-S473 Rabbit mAb | Abclonal | Cat# AP1208 |
| Phospho-AMPKα1-T183+AMPKα2-T172 Rabbit mAb | Abclonal | Cat# AP1441 |
| Phospho-Histone H2AX-S139 Rabbit mAb | Abclonal | Cat# AP0687 |
| Phospho-HSL (Ser563) Antibody | Cell Signaling Technology | Cat# 4139 |
| Phospho-mTOR-S2448 Rabbit mAb | Abclonal | Cat# AP0115 |
| Phospho-Perilipin-1 (Ser522) Antibody | Affinity Biosciences | Cat# AF3834 |
| PPARγ Rabbit pAb | Abclonal | Cat# A0270 |
| **Chemicals, Peptides, and Recombinant Proteins** | | |
| 2-NBDG | Cayman | Cat# 11046-1mg |
| A922500 | TargetMol | Cat# T6365 |
| AICAR | TargetMol | Cat# T1477 |
| Annexin V Binding Buffer | BD Biosciences | Cat# 556454 |
| APC Annexin V | BD Biosciences | Cat# 550474 |
| Atglistatin | TargetMol | Cat# T1875 |
| Avasimibe | Cayman | Cat# 18129 |
| BODIPY 493/503 | Cayman | Cat# 25892 |
| C11 BODIPY 581/591 | Cayman | Cat# 27086 |
| Cell Counting Kit-8 (CCK-8) | Beyotime | Cat# C0038 |
| ChamQ SYBR qPCR Master Mix | Vazyme | Cat# Q321-02 |
| Collagenase IV | Gibco | Cat# 17104-019 |
| DAPI | Beyotime | Cat# C1005 |
| DMSO | MCE | Cat# HY-Y0320 |
| DNase I | Sigma-Aldrich | Cat# 11284932001 |
| FITC Annexin V | BD Biosciences | Cat# 556419 |
| Forskolin | TargetMol | Cat# T2939 |
| GW9662 | Beyotime | Cat# SC9123 |
| HiScript III RT SuperMix for qPCR | Vazyme | Cat# R323-01 |
| Histopaque®-1077 | Sigma-Aldrich | Cat# 10771 |
| Histopaque®-1119 | Sigma-Aldrich | Cat# 11191 |
| Human DiI-Oxidized Low Density Lipoprotein | ThermoFisher | Cat# L34358 |
| Lipofectamine™ 2000 | Invitrogen | Cat# 11668019 |
| LY294002 | TargetMol | Cat# T2008 |
| MitoSOX Red Mitochondrial Superoxide Indicator | Abclonal | Cat# RM02822 |
| Mito-TEMPO | TargetMol | Cat# T19428 |
| MK-2206 | TargetMol | Cat# T1952 |
| Nycodenz | Serumwerk Bernburg AG | Cat# 18003 |
| Oil red O | Solarbio | Cat# G1260 |
| Oleic acid | TargetMol | Cat# T2O2668 |
| Oxaliplatin | TargetMol | Cat# T0164 |
| PF06424439 | TargetMol | Cat# T12425 |
| Propidium Iodide Staining Solution | BD Biosciences | Cat# 556463 |
| ProstaglandinE2(PGE2) | TargetMol | Cat# T5014 |
| Rapamycin | Cayman | Cat# 13346 |
| Recombinant human EGF | Beyotime | Cat# P5552 |
| Recombinant human GM-CSF (CSF2) | Beyotime | Cat# P5286 |
| Recombinant human IFN-γ | Beyotime | Cat# P5664 |
| Recombinant human IGF-2 | Beyotime | Cat# P5538 |
| Recombinant human IL-1 beta | novoprotein | Cat# GMP-CG93 |
| Recombinant human IL-13 | Beyotime | Cat# P5178 |
| Recombinant human IL-17/IL-17A | Novus | Cat# NBP2-35040 |
| Recombinant human IL-33 | Beyotime | Cat# P5237 |
| Recombinant human IL-4 | R&D Systems | Cat# 204-IL-010 |
| Recombinant human Transforming Growth Factor β-1/TGFB1 | Absin | Cat# abs04204 |
| Recombinant human Wnt3A | Sigma-Aldrich | Cat# H17001 |
| Recombinant murine IL-33 | R&D Systems | Cat# 3626-ML-010/CF |
| Red blood cell lysis buffer | Solarbio | Cat# R1010 |
| Sulfosuccinimidyl oleate | TargetMol | Cat# T13036L |
| Tribromoethyl alcohol | Sigma-Aldrich | Cat# T48402 |
| TRIzol | Invitrogen | Cat# 15596018CN |
|  |  |  |
| **Critical Commercial Assays** | | |
| BeyoClick™ EdU Cell Proliferation Kit with Alexa Fluor 555 | Beyotime | Cat# C0075L |
| CD3/CD28 Streptamer® Kit, human | IBA Life Sciences | Cat# 6-8900-050 |
| CellTiter-Lumi™ Steady Luminescent Cell Viability Assay Kit | Beyotime | Cat# C0065 |
| CellTrace CFSE Cell Proliferation Kits | Invitrogen | Cat# C34570 |
| Fatty Acid Oxidation (FAO) Assay Kit | BMR | Cat# E-141 |
| Fixation/Permeabilization Solution Kit with BD GolgiStop | BD Biosciences | Cat# 554715 |
| Free fatty Acids (FFA) Content Assay Kit | Solarbio | Cat# BC0595 |
| Lipid Extraction Kit | Cell Biolabs | Cat# STA-612 |
| Mitochondrial membrane potential assay kit with JC-1 | Beyotime | Cat# C2006 |
| Senescence beta-Galactosidase Staining Kit | Cell Signaling Technology | Cat# 9860 |
| Triglyceride (TG) Content Assay Kit | Solarbio | Cat# BC0625 |
| **Software and Algorithms** | | |
| Adobe Illustrator 2022 | Adobe | https://www.adobe.com/de/products/illustrator.html |
| CytExpert | BECKMAN | N/A |
| Fiji, ImageJ | ImageJ | https://imagej.net/Downloads |
| FlowJo V10 | FlowJO | https://www.flowjo.com/solutions/flowjo/downloads/ |
| GraphPad Prism 8 | GraphPad | https://www.graphpad.com/scientific-software/prism/ |
